# Genomic transcriptional profiling identifies a candidate blood biomarker signature for the diagnosis of septicemic melioidosis

**DOI:** 10.1186/gb-2009-10-11-r127

**Published:** 2009-11-10

**Authors:** Rungnapa Pankla, Surachat Buddhisa, Matthew Berry, Derek M Blankenship, Gregory J Bancroft, Jacques Banchereau, Ganjana Lertmemongkolchai, Damien Chaussabel

**Affiliations:** 1 Department of Clinical Immunology, Centre for Research and Development of Medical Diagnostic Laboratories, Faculty of Associated Medical Sciences, Khon Kaen University, 123 Mittraparp Road, Khon Kaen, 40002, Thailand; 2 Baylor-National Institute of Allergy and Infectious Diseases (NIAID), Cooperative Center for Translational Research on Human Immunology and Biodefense, Baylor Institute for Immunology Research and Baylor Research Institute, 3434 Live Oak St, Dallas, Texas, 75204, USA; 3 Division of Immunoregulation, National Institute for Medical Research, The Ridgeway, Mill Hill, London, NW7 1AA, UK; 4 Institute for Health Care Research and Improvement, Baylor Health Care System, 8080 N. Central Expressway Suite 500, Dallas, Texas, 75206, USA; 5 Department of Infectious and Tropical Diseases, London School of Hygiene and Tropical Medicine, Keppel St, London, WC1E 7HT, UK

## Abstract

**Background:**

Melioidosis is a severe infectious disease caused by *Burkholderia pseudomallei*, a Gram-negative bacillus classified by the National Institute of Allergy and Infectious Diseases (NIAID) as a category B priority agent. Septicemia is the most common presentation of the disease with a 40% mortality rate even with appropriate treatments. Better diagnostic tests are therefore needed to improve therapeutic efficacy and survival rates.

**Results:**

We have used microarray technology to generate genome-wide transcriptional profiles (>48,000 transcripts) from the whole blood of patients with septicemic melioidosis (n = 32), patients with sepsis caused by other pathogens (n = 31), and uninfected controls (n = 29). Unsupervised analyses demonstrated the existence of a whole blood transcriptional signature distinguishing patients with sepsis from control subjects. The majority of changes observed were common to both septicemic melioidosis and sepsis caused by other infections, including genes related to inflammation, interferon-related genes, neutrophils, cytotoxic cells, and T-cells. Finally, class prediction analysis identified a 37 transcript candidate diagnostic signature that distinguished melioidosis from sepsis caused by other organisms with 100% accuracy in a training set. This finding was confirmed in 2 independent validation sets, which gave high prediction accuracies of 78% and 80%, respectively. This signature was significantly enriched in genes coding for products involved in the MHC class II antigen processing and presentation pathway.

**Conclusions:**

Blood transcriptional patterns distinguish patients with septicemic melioidosis from patients with sepsis caused by other pathogens. Once confirmed in a large scale trial this diagnostic signature might constitute the basis of a differential diagnostic assay.

## Abstract 2

A diagnostic signature for sepsis caused by *Burkholderia pseudomallei* infection was identified from transcriptional profiling of the blood of septicemia patients.

## Background

Melioidosis is an infectious disease caused by the Gram-negative bacillus *Burkholderia pseudomallei*. The disease is endemic in northern Australia, Southeast Asia, and northeast Thailand, where it is a common cause of community-acquired sepsis [1,2]. Cases of melioidosis have also been reported from other regions around the world [3]. In Thailand, the incidence rate of melioidosis was estimated as 4.4 cases per 100,000 individuals, but melioidosis cases are under-reported due to a lack of adequate laboratory testing [1,4]. The disease is the leading cause of community-acquired septicemia in northeast Thailand [5]. The common clinical manifestation of melioidosis at initial presentation is febrile illness with pneumonia, which makes it difficult to distinguish from other infections [1,6]. However, in contrast to other infections, the majority of melioidosis patients develop sepsis rapidly after presentation, and the disease has a mortality rate of 40% despite appropriate treatment [6].

Definitive diagnosis requires isolation of *B. pseudomallei *from clinical specimens [1,7-9]. However, the rate of positive cultures is low and it may take up to a week to confirm a microbiological diagnosis of melioidosis, which can delay the initiation of appropriate therapy [1,10-12]. Antibody detection by indirect hemagglutination assay is faster than culture but lacks sensitivity and specificity, especially when used in an endemic area since most of the population is seropositive [1]. Amplification approaches to detect pathogen-specific genes by PCR have similarly shown variable specificity and sensitivity [7-9]. Missed or delayed diagnosis may have dire consequences since several antibiotics commonly used for Gram-negative septicemia are ineffective against *B. pseudomallei *[1,3,13]. It has been reported that faster diagnosis of other bloodstream infections permits earlier implementation of appropriate antimicrobial therapy and reduces mortality [14]. Animal models support the notion that an earlier diagnosis of melioidosis leads to an improved disease outcome, with increased survival observed when *B. pseudomallei*-infected mice are treated with the appropriate antibiotics within 24 hours post-infection [15]. Thus, there is an urgent need for improved, rapid diagnostic tests for septicemic melioidosis and indicators of clinical severity [1,6,10]. Furthermore, *B. pseudomallei *has been classified as a category B agent of bioterrorism by the US Centers for Disease Control and Prevention and the National Institute of Allergy and Infectious Diseases (NIAID) due to its ability to initiate infection via aerosol contact; the rapid onset of sepsis following the development of symptoms and the high mortality rate even with medical treatment [16]. Taken together, these facts delineate the importance of developing novel tools for the rapid and definitive diagnosis of *B. pseudomallei *infection.

Microarray-based profiling of tumoral tissue has proved instrumental for the discovery of transcriptional biomarker signatures in patients with cancer [17]. The immune status of a patient can be assessed through the profiling of peripheral blood, which constitutes an accessible source of immune cells that migrate to and from sites of infection, and are exposed to pathogen as well as host-derived factors released in the circulation. Furthermore, through the analysis of whole blood it is possible to measure transcriptional responses caused by disease with minimal sampling bias or *ex vivo *manipulation. The use of gene expression microarrays as a tool to study the expression profiles of human blood has been reported in systemic autoimmune diseases and infectious diseases, including malaria, acute dengue hemorrhagic fever, febrile respiratory illness, and influenza A virus or bacterial infections [18-22]. In addition, previous studies have shown that microarray-based approaches allow researchers to identify blood expression profiles restricted to sepsis [23-25]. In the context of the present study, we have used a microarray-based approach to generate blood transcriptional profiles of septic patients who were recruited in northeast Thailand. After establishing a blood signature of sepsis, we developed a candidate biomarker signature that distinguishes *B. pseudomallei *from other infectious agents causing septicemia.

## Results

### Patient characteristics

**Figure 1:**
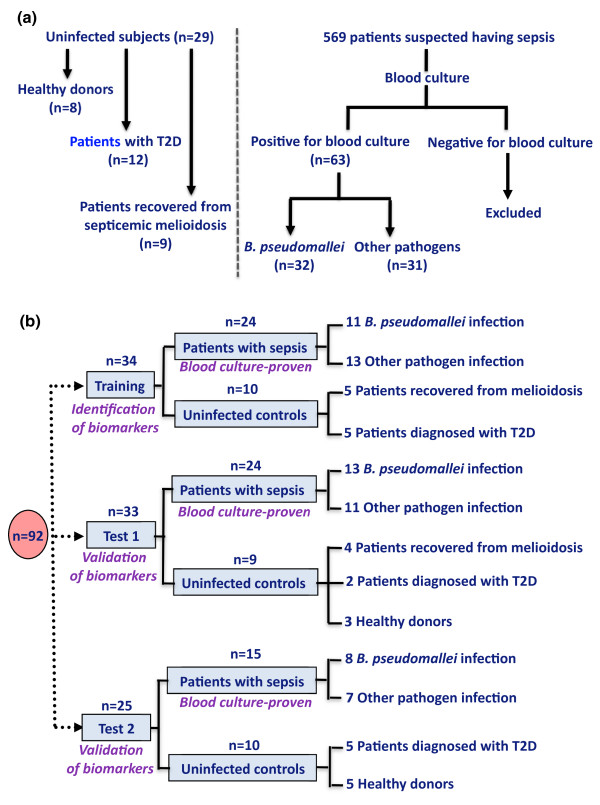
Subject enrolment and study design. **(a) **Recruitment strategy. A total of 598 subjects consisting of 29 uninfected controls and 569 patients diagnosed with sepsis were recruited in this study. Of the patients diagnosed with sepsis (569 subjects), only those with positive blood cultures (63 subjects) were included for further study. Subjects who had no signs of infection (29 subjects) were also recruited to constitute an uninfected control group, including healthy donors, patients diagnosed with T2D, and patients who had recovered from melioidosis. Subjects for this latter group could not be recruited in our second validation set. **(b) **Study design. The diagram depicts the composition of the training and independent test sets. Of 92 subjects enrolled in this study, 34 were assigned to the training set, 33 were assigned to the test set 1, and 25 were assigned to the test set 2. T2D, type 2 diabetes.

A total of 598 subjects consisting of 29 uninfected controls and 569 patients diagnosed with sepsis were enrolled in this study and all subjects were Asian (Figure 1a). Of these 569 patients, 63 had positive blood cultures (32 grew *B. pseudomallei *and 31 grew other organisms) and were thus selected for microarray analysis. Meanwhile, 29 uninfected controls recruited in this study were 8 healthy donors, 12 patients with type 2 diabetes (T2D) and 9 patients who had recovered from melioidosis. Whole blood samples collected from these 29 uninfected controls and 63 septic patients were extracted for RNA in 3 separated experiments: the first set (34 samples) was assigned to a training set used for discovery; the second set (33 samples) was assigned to a first test set to independently evaluate the performance of candidate markers; and the third set (25 samples) was assigned to a second independent test set for further validation (Figure 1b and Table 1).

**Table 1:** Demographic, clinical and microbiological data of 92 subjects

	Septicemic melioidosis	Other sepsis	Recovery	Type 2 diabetes	Healthy
**Training set (n = 34)**					
Number of subjects	11	13	5	5	
Mean age in years (range)	54 (41-70)	56 (37-74)	46 (41-64)	40 (39-68)	
Sex (male/female)	7/4	4/9	3/2	1/4	
Survivors/non-survivors	6/5	11/2			
Organisms (n)	*B. pseudomallei *(11)	*A. baumannii *(1)			
		*Corynebacterium *spp. (2)			
		*C. albicans *(3)			
		*E. coli *(3)			
		*Salmonella *serotype B (1)			
		*S. aureus *(1)			
		*Salmonella *spp. (1)			
		Non-group A or B *Streptococcus *(1)			
**Independent test set 1 (n = 33)**					
Number of subjects	13	11	4	2	3
Mean age in years (range)	50 (18-70)	56 (37-70)	50 (39-64)	49 (48-50)	38 (35-43)
Sex (male/female)	11/2	6/5	3/1	0/2	0/3
Survivors/non-survivors	12/1	6/5			
Organisms (n)	*B. pseudomallei *(13)	*Coagulase-negative staphylococci *(6)*			
		*E. coli *(1)			
		*Enterococcus *spp. (1)			
		*S. aureus *(1)			
		*K. pneumoniae *(1)			
		*S. pneumoniae *(1)			
**Independent test set 2 (n = 25)**					
Number of subjects	8	7		5	5
Mean age in years (range)	47 (40-56)	61 (43-81)		57 (50-71)	44 (37-67)
Sex (male/female)	4/4	2/5		0/5	3/2
Survivors/non-survivors	3/5	5/2		5/0	5/0
Organisms (n)	*B. pseudomallei *(8)	*A. hydrophila *(1)^†^			
		*Corynebacterium *spp. (1)			
		*E. coli *(2)^†^			
		*S. aureus *(1)			
		*Enterococcus *spp. (1)			
		*E. faecium *(1)			

*Three in six patients were positive in two sets of blood cultures. ^†^Patients were positive in two sets of blood cultures.

The training set is composed of 34 samples: 24 patients with sepsis, all with positive blood cultures, including 11 patients with septicemic melioidosis; 13 patients with sepsis due to other organisms (1 *Acinetobacter baumannii*, 2 *Corynebacterium *spp., 3 *Candida albicans*, 3 *Escherichia coli*, 1 *Salmonella *serotype B, 1 *Salmonella *spp., 1 *Staphylococcus aureus*, and 1 non-group A or B *Streptococcus*); and 10 subjects from the same endemic area recruited as non-infected controls. These non-infected controls comprised 5 patients with T2D, a risk factor for melioidosis, and 5 patients with melioidosis who have recovered after complete treatment, and been followed up for at least 20 weeks without any sign of infection; 3 out of these 5 subjects were diabetic. Demographic, clinical and microbiological data are available in Table 2 and Additional data file 1.

**Table 2:** Characteristics of patients in the training set

Sample ID	Age (years)	Sex	Bacterial isolation	Antibiotherapy before blood collection	Underlying diseases	Survival
**Other sepsis (n = 13)**						
I001*	52	Male	*Streptococcus *non-group A or B	Ceftriaxone	-	Non-survivor
I002^†‡^	52	Female	*A. baumannii*	Ceftazidime, bactrim	T2D, CRF, lung edema	Survivor
I004* ^‡^	45	Male	*Salmonella *serotype B	Cloxacillin, ceftriaxone	T2D, arthritis	Survivor
I006* ^§^	37	Male	*C. albicans*	Ceftriaxone, sulperazone, bactrim	HIV infection, tuberculosis	Survivor
I007*	73	Female	*Corynebacterium *spp.	-	NSAID-induced GI bleeding	Non-survivor
I008^†¶^	70	Female	*E. coli*	Bactrim, ceftazidime	T2D	Survivor
I009*	52	Female	*S. aureus*	Ceftazidime, cloxacillin	T2D, knee abscess	Survivor
I010^†‡¥^	72	Female	*E. coli*	Ceftriaxone	T2D, CRF	Survivor
I011* ^¶^	38	Female	*E. coli*	-	HCV infection	Survivor
I012* ^§^	69	Female	*C. albicans*	Ceftazidime	RF	Survivor
I013*	74	Female	*Corynebacterium *spp.	Ceftazidime, clarithromycin	Chronic heart failure, COPD	Survivor
I014*	54	Female	*Salmonella *spp.	Ceftriaxone, ceftazidime, levofloxacin	T2D, endometrial cancer, ITP	Survivor
I015* ^§^	41	Male	*C. albicans*	Ceftazidime	HIV infection	Survivor
						
**Septicemic melioidosis (n = 11)**						
M001*	68	Male	*B. pseudomallei*	Ceftazidime, bactrim	Chronic heart failure, COPD	Non-survivor
M002*	43	Female	*B. pseudomallei*	Ceftriaxone, ceftazidime	T2D	Survivor
M003*	55	Male	*B. pseudomallei*	Ceftazidime	-	Non-survivor
M006*	46	Male	*B. pseudomallei*	Ceftriaxone	T2D, chirrosis	Non-survivor
M007*	50	Male	*B. pseudomallei*	Ceftazidime, tazocin	Lung cancer	Survivor
M008*	70	Female	*B. pseudomallei*	Ceftazidime, bactrim	T2D	Non-survivor
M009*	48	Female	*B. pseudomallei*	Sulperazone	T2D	Survivor
M010*	48	Male	*B. pseudomallei*	Ceftriaxone, ceftazidime, doxycycline	T2D	Survivor
M012*	56	Male	*B. pseudomallei*	Sulperazone, bactrim, cetazidime	T1D, ARF	Survivor
M014*	65	Female	*B. pseudomallei*	Cloxacilin, ceftazidime	T2D, chirrosis	Non-survivor
M015*	41	Male	*B. pseudomallei*	Bactrim, ceftazidime	-	Survivor

*Community-acquired septicemia; ^†^hospital-acquired septicemia;^‡^mechanical ventilation;^§^taken immunosuppressive;^¶^urinary catheterized drugs; ^¥^blood transfused. ARF, acute renal failure; COPD, chronic obstructive pulmonary disease; CRF, chronic renal failure; GI, gastrointestinal tract; NSAID, non-steroidal anti-inflammatory drug; RF, renal failure; T2D, type 2 diabetes; TP, idiopathic thrombocytopenic purpura.

The first independent test set (test set 1) is composed of 33 samples: 24 patients with sepsis, including 13 patients with septicemic melioidosis, and 11 patients with sepsis and isolation of other organisms (6 coagulase-negative staphylococci, 1 *S. aureus*, 1 *Streptococcus pneumoniae*, 1 *Klebsiella pneumoniae*, 1 *Enterococcus *spp., and 1 *E. coli*); and 9 control samples, including 4 patients who recovered from melioidosis, 2 patients with T2D, and 3 healthy donors from the same endemic area. Demographic, clinical and microbiological data are available in Table 3 and Additional data file 1.

**Table 3:** Characteristics of patients in the independent test set 1

Sample ID	Age (years)	Sex	Bacterial isolation	Antibiotherapy before blood collection	Underlying diseases	Survival
**Other sepsis (n = 11)**						
I016* ^†^	61	Female	Coagulase-negative staphylococci	Ceftazidime, bactrim, Sulperazole	Hematemesis	Survivor
I017* ^‡§^	50	Male	Coagulase-negative staphylococci	Ceftriaxone, ceftazidime, doxycycline, cloxacillin	Acute pancreatitis, nephrotic syndrome	Survivor
I018^§¶¥^	57	Male	Coagulase-negative staphylococci^#^	Vancomycin	T2D, CRF	Survivor
I019^¤^	58	Female	*Staphylococcus aureus*	Cloxacillin, ceftazidime	T2D, wound	Survivor
I020^¶¥^	66	Female	Coagulase-negative staphylococci^#^	Ceftazidime, ceftriaxone	T2D, ARF, tuberculosis	Non-survivor
I021^¶^	54	Female	*Enterococcus *spp.	Ceftazidime, cloxacilin	T2D, abscess	Non-survivor
I022^§¶^	37	Male	Coagulase-negative staphylococci^#^	Ceftriaxone, ceftazidime	T2D, ARF	Non-survivor
I023^¶¤^	70	Female	*E. coli*	Doxycycline, ceftazidime	T2D	Non-survivor
I024^¶¥^	56	Male	Coagulase-negative staphylococci	Meropenem, ceftazidime	T2D, RF	Survivor
I025*	50	Male	*S. pneumoniae*	Ceftriaxone, meropenem	T2D	Non-survivor
I026^¶^	57	Male	*K. pneumoniae*	Ceftriaxone, ceftazidime, bactrim	T2D	Survivor
						
**Septicemic melioidosis (n = 13)**						
M016^¶^	39	Male	*B. pseudomallei*	Ceftazidime, bactrim, doxycycline	T2D	Survivor
M017^¶^	52	Female	*B. pseudomallei*	Norfloxacin, ceftazolin	T2D	Survivor
M020^¶^	61	Male	*B. pseudomallei*	Ceftriaxone, doxycycline, ceftazidime	-	Survivor
M021^¶^	56	Female	*B. pseudomallei*	Ceftriaxone, ceftazidime	T2D	Survivor
M022^¶^	18	Male	*B. pseudomallei*	Ceftazidime, cactrim	T2D	Survivor
M023^¶^	63	Male	*B. pseudomallei*	Bactrim, ceftazidime	T2D	Survivor
M024^¶^	44	Male	*B. pseudomallei*	Meropenem	T2D, RF	Survivor
M025^¶^	57	Male	*B. pseudomallei*	Ceftazidime	T2D	Survivor
M026^¶^	48	Male	*B. pseudomallei*	Ceftazidime, doxycycline, bactrim	T2D	Survivor
M027^¶^	44	Male	*B. pseudomallei*	Ceftriaxone, ceftazidime, meropenem	ARF	Survivor
M028^¶^	70	Male	*B. pseudomallei*	Ceftazidime, levofloxacin, bactrim	T2D	Survivor
M029^¶^	50	Male	*B. pseudomallei*	Ceftriaxone, ceftazidime	CRF	Non-survivor
M030^¶^	44	Male	*B. pseudomallei*	Ceftazidime, ceftriazone	T2D, tuberculosis	Survivor

*Hospital-acquired septicemia;^†^long hospitalization;^‡^taken immunosuppressive drugs;^§^dialysis; ^¶^community-acquired septicemia; ^¥^mechanical ventilation; ^¤^wounds. ^#^Positive by two sets of blood cultures. ARF, acute renal failure; CRF, chronic renal failure; RF, renal failure; T2D, type 2 diabetes.

The second independent test set (test set 2) is composed of 25 samples: 15 patients with sepsis, including 8 patients with septicemic melioidosis, and 7 patients with sepsis and isolation of other organisms (2 *E. coli*, 1 *S. aureus*, 1 *Corynebacterium *spp., 1 *Enterococcus *spp., 1 *Enterococcus faecium*, and 1 *Aeromonas hydrophila*); and 10 control samples, including 5 patients with T2D and 5 healthy donors. The demographic, clinical data and microbiological data are available in Table 4 and Additional data file 1.

**Table 4:** Characteristics of patients in the independent test set 2

Sample ID	Age (years)	Sex	Bacterial isolation	Antibiotherapy before blood collection	Underlying diseases	Survival
**Other sepsis (n = 7)**						
I027*	64	Female	*E. coli*^†^	Fortum, ceftriaxone	UGIB	Non-survivor
I028^‡^	81	Female	*Corynebacterium *spp.	Ceftriaxone, fortum, clindamycin	T2D	Survivor
I029^‡^	74	Female	*S. aureus*	Fortum, ceftriaxone, tazocin	Asthma, emphysema, ARF	Survivor
I031*	48	Male	*Enterococcus spp.*	Fortum	Urinary tract infection	Survivor
I032*	54	Female	*E. faecium*	Fortum, tazocin	T2D, respiratory failure	Non-survivor
I033*	63	Female	*E. coli*^†^	Tazocin, ceftriaxone, fortum	T2D, ovarian cancer	Survivor
I034*	43	Male	*A. hydrophila*^†^	Tazocin	-	Survivor
						
**Septicemic melioidosis (n = 8)**						
M031*	49	Male	*B. pseudomallei*	Fortum, bactrim, tazocin	T2D	Non-survivor
M032*	54	Male	*B. pseudomallei*	Fortum, doxycycline, sulperazone	T2D	Non-survivor
M033*	44	Male	*B. pseudomallei*	Fortum, sulperazone, bactrim, ciprofloxacin	T2D	Survivor
M034*	40	Female	*B. pseudomallei*	Fortum, bactrim, ceftazidime, ceftriaxone	T2D	Survivor
M035*	56	Male	*B. pseudomallei*	Ceftriaxone, ceftazidime, fortum	COPD, T2D	Non-survivor
M036*	41	Female	*B. pseudomallei*	Ceftriaxone, ceftazidime	T2D	Non-survivor
M037*	42	Female	*B. pseudomallei*	Bactrim, fortum, cloxacillin	T2D	Survivor
M038*	49	Female	*B. pseudomallei*	Ceftriaxone, fortum, ceftazidime, levofloxacin	-	Non-survivor

*Community-acquired septicemia; ^‡^hospital-acquired septicemia. ^†^Positive by two sets of blood cultures. ARF, acute renal failure; COPD, chronic obstructive pulmonary disease; T2D, type 2 diabetes; UGIB, upper gastrointestinal bleeding.

All groups were similar in terms of race. There was no statistically significant difference in age among the data sets and disease status groups (ANOVA overall F test, *P*-value = 0.0884). There was also no statistically significant difference in gender among the data sets and disease groups (Fisher's exact test with Bonferroni correction, all *P*-values ≥0.274). No statistically significant differences were found between whole blood samples collected from patients with septicemic melioidosis and patients with sepsis and isolation of other organisms in the training and the two test sets concerning the total leukocyte, platelet, neutrophil, lymphocyte, and monocyte blood cell counts (Table S1 in Additional data file 2). Out of 92 subjects, 58 were diagnosed with T2D (63%), a well-documented risk factor for melioidosis. Of these 58 diabetic subjects, 17 were uninfected controls whereas 41 were septic patients. Pneumonia was found in 20 patients with melioidosis (63%) and in 12 of the septic patients with infections caused by other organisms (39%). In addition, 4 out of 63 patients with sepsis were immunocompromised, including 2 patients under immunosuppressive therapy and 2 patients with underlying HIV infection.

### Blood transcriptional profiles of septic patients and healthy or diabetic controls are distinct

We first wanted to determine whether transcriptional profiles of septicemic patients were distinct from those of healthy individuals and individuals with T2D. We started by carrying out unsupervised analyses that consist in exploring molecular signatures in a dataset without *a priori *knowledge of sample phenotype or grouping. Blood profiles from the training dataset (24 septicemic patients and 10 controls) were first subjected to this analysis. Filters were applied to remove transcripts that are not detected in at least 10% of all samples (detection *P*-value < 0.01), and that are expressed at similar levels across all conditions, that is, present little deviation from the median intensity value calculated across all samples (less than 2-fold and 200 intensity units from the median; see Materials and method section for details). From a total of 48,701 probes arrayed on the Illumina Hu6 V2 beadchip, 16,400 transcripts passed the detection filter and 2,785 transcripts passed both filters.

**Figure 2:**
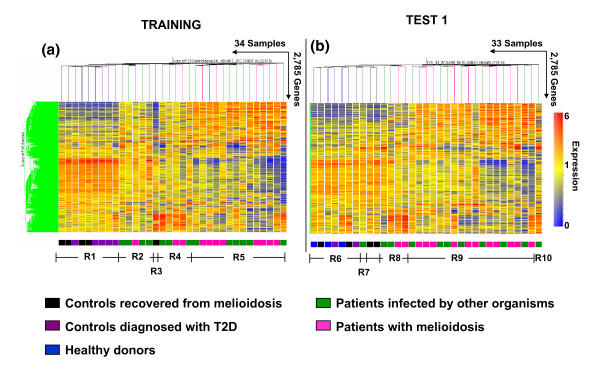
Unsupervised hierarchical clustering of blood transcriptional profiles of septic patients. Transcripts with 2-fold over- or under-expression compared with the median of all samples and differential expression values greater than 200 from the median for each gene in at least 2 samples in the training set were selected for unsupervised analysis (n = 2,785 transcripts). **(a) **A heatmap resulting from hierarchical clustering of transcripts and conditions (subjects) was generated for the training set. **(b) **The same gene tree of these 2,785 transcripts was then used to generate a heatmap for the first independent test set (test set 1) using hierarchical clustering of conditions as before. The color conventions for heatmaps are as follows: red indicates over-expressed transcripts; blue represents underexpressed transcripts; and yellow indicates transcripts that do not deviate from the median. Study group is marked as follows: patients with melioidosis are indicated by pink rectangles; patients with sepsis due to other organisms by green rectangles; uninfected controls who recovered from melioidosis by black rectangles; T2D patients by purple rectangles; and healthy donors by blue rectangles. This unsupervised hierarchical clustering of blood transcriptional profiles was observed to segregate into five distinct regions in both training (regions R1 to R5) and test sets (regions R6 to R10).

This set of 2,785 transcripts was used in an unsupervised hierarchical clustering analysis where transcripts are ordered horizontally and samples (conditions) vertically, according to similarities in expression patterns (Figure 2a). The resulting heatmap reveals the molecular heterogeneity of this sample set. The molecular classification obtained through hierarchical clustering is then compared with phenotypic classification of the samples: out of the ten uninfected controls, nine samples were clustered together on a branch of the condition tree (region R1) that is distinct from that of septicemic patients (regions R2, R4, and R5). One outlying uninfected control clustered together with septicemic patients (sample R001 in region R3). The expression pattern for this outlying sample appeared nonetheless distinct from that of septicemia and it was excluded from subsequent class comparison analyses.

We further explored the molecular heterogeneity of this sample set through principal component analysis (PCA; Figure S1 in Additional data file 2). PCA is a useful tool to reduce the dimension and complexity of microarray data. The 2,785 most variable transcripts selected above were decomposed into 7 principal components (PCs). The first 3 major PCs accounted for 40.1% (PC1), 18.2% (PC2), and 6.2% (PC3) of the variability observed for these conditions. This three-dimensional plot confirmed the segregation of uninfected controls from septicemic patients with the exception of the same outlying sample (sample R001).

We repeated this analysis for the independent test set 1 (n = 33) using the same 2,785 transcripts previously identified in the analysis of the training set. Once again, unsupervised hierarchical clustering revealed distinctive transcriptional profiles separating uninfected controls (region R6) from patients with sepsis (regions R8, R9, and R10) (Figure 2b). Thus, the results of the unsupervised analysis clearly established the existence of a robust blood transcriptional signature in the context of sepsis that is distinct from that of uninfected controls. Indeed, the sample grouping (separation of healthy controls and T2D compared to sepsis) and lack thereof (non-separation of healthy controls compared to T2D) observed following unsupervised hierarchical clustering (Figure 2) and PCA (Figure S1 in Additional data file 2) indicates that the transcriptional profile of T2D patients is more similar to healthy controls than to patients with sepsis. This suggests that the transcriptional perturbation induced by melioidosis or sepsis is of such a magnitude as to render any such effect from T2D undetectable in comparison.

To examine the biological significance of the 2,785 transcript signature, we extracted annotations from the Database for Annotation, Visualization and Integrated Discovery (DAVID) using Expression Analysis Systematic Explorer (EASE). The major biological Gene Ontology term enrichments categorized from these 2,785 transcripts are shown in Figure S2 in Additional data file 2. This analysis associated transcripts with several biological categories, including defense response (*CD55*, *CD59*, *LTF*, *TLR2*), immune system process (*GBP6*, *HLA-A*, *HLA-DMA*, *BCL2*), response to stress (*ZAK*, *GP9*, *DUSP1*, *PTGS1*), and inflammatory response (*CFH*, *TLR4*, *IL1B*, *SERPING1*) [26].

Next, we identified and independently validated sets of transcripts differentially expressed between uninfected controls and patients with sepsis by carrying out direct comparison between these two groups (supervised analysis). Starting from the list of genes present in at least 10% of samples defined above (n = 16,400), we performed statistical comparisons (Welch *t*-test, *P *< 0.01) with three different stringencies of multiple testing corrections and returned sets of transcripts for which expression levels were significantly different between the two study groups (Table S2 and Figure S3 in Additional data file 2). Using the most stringent Bonferroni correction for controlling type I error, 2,733 transcripts were found differentially expressed between these two groups. Applying a more liberal correction, the Benjamini and Hochberg false discovery rate, to the analysis yielded an expanded list of 7,377 transcripts differentially expressed between these two groups (false discovery rate = 1%). Finally, performing the statistical analysis without any multiple testing correction yielded 8,096 differentially expressed transcripts with 164 transcripts expected to be positive by chance alone. These 3 transcriptional signatures identified using different statistical stringencies were then validated independently in the first test set composed of 9 uninfected controls and 24 patients with sepsis. We found that hierarchical clustering discriminated perfectly between the two groups in this independent test set when using the probes identified with the Bonferroni correction (Figure S3f in Additional data file 2). Class prediction analysis further confirmed these results since a set of 10 predictors gave over 95% in sensitivity and specificity in the training set (K-nearest neighbors; leave-one-out cross-validation) and 96% sensitivity and 89% specificity in the first independent test set (Table S3 in Additional data file 2).

In conclusion, these results demonstrate that whole blood transcriptional profiles in patients with sepsis and in non-infected controls are distinct.

### Blood transcriptional profiles of septic patients are heterogeneous

**Figure 3:**
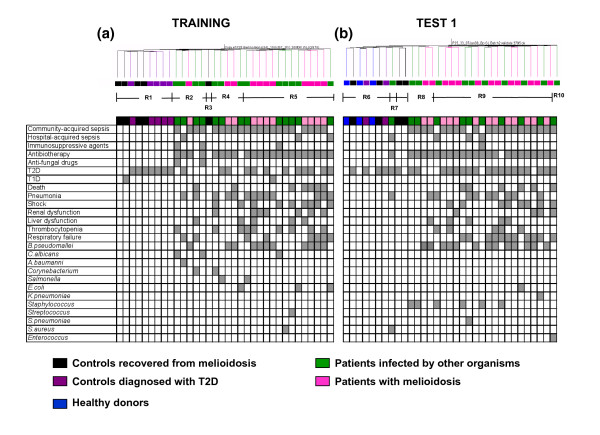
Comparison of phenotypic and clinical information with unsupervised condition clustering. The distribution of subjects who were defined as community-acquired or nosocomial septicemia, given antibiotics before blood collection (Antibiotherapy), diagnosed with T1D or T2D, organ dysfunction, pneumonia, and microbial diagnosis is indicated on a grid aligned against the hierarchical condition tree generated through unsupervised clustering (Figure 2) for both **(a) **training and **(b) **test set 1.

While the signature of sepsis is clearly distinct from that of uninfected controls, unsupervised analyses revealed that it was also heterogeneous. Indeed, distinct patterns are discernable on the heatmaps generated from the training set (Figure 2a, regions R2, R4, and R5) and test set 1 (Figure 2b, regions R8, R9, and R10). This heterogeneity cannot be explained by etiological differences since the pathogen species identified are distributed among the different regions (R2: 2 *C. albicans*, 1 *A. baumannii*, 1 *Corynebacterium *spp., and 1 *B. pseudomallei*; R4: 1 *Corynebacterium *spp., 1 *Salmonella *serotype B, 1 *E. coli*, and 2 *B. pseudomallei*; R5: 1 *Salmonella *spp., 1 *S. aureus*, 1 *Streptococcus *non group A or B, 1 *C. albicans*, 2 *E. coli*, and 8 *B. pseudomallei*; R8: 2 coagulase-negative staphylococci, 2 *B. pseudomallei*; R9: 4 coagulase-negative staphylococci, 1 *S. pneumoniae*, 1 *E. coli*, 1 *K. pneumoniae*, 11 *B. pseudomallei*; R10: 1 *Enterococcus *spp.), nor can it be attributed to differences in treatment, co-morbidity or pulmonary involvement (Figure 3a, b).

**Figure 4:**
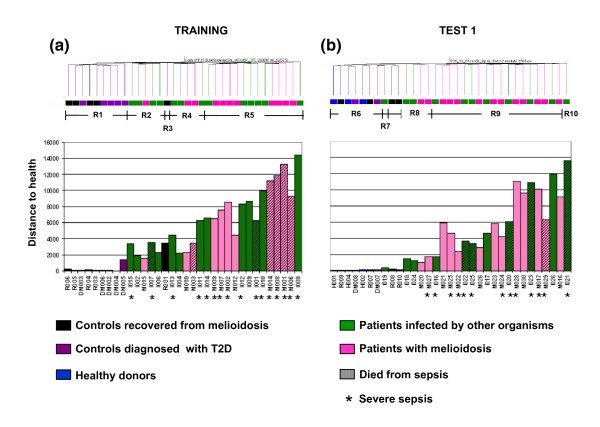
Comparison of molecular distances from baseline samples with unsupervised condition clustering. The list of 2,785 transcripts identified in the unsupervised analysis (Figure 2) was used to compute the 'molecular distance' between samples from patients with sepsis and uninfected control samples. **(a, b) **Region R1 for the training set (a) and R6 for the first test set (b) were used as the baseline uninfected controls for all comparisons. Molecular distances for individual subjects are indicated on a histogram that is aligned against the hierarchical condition tree generated through unsupervised clustering (Figure 2). Study group is marked as follows: patients with melioidosis are indicated by pink rectangles; patients with sepsis due to other organisms by green rectangles; uninfected controls who recovered from melioidosis by black rectangles; T2D patients by purple rectangles; and healthy donors by blue rectangles. Patients who died from sepsis are indicated by diagonal shading within the bars. Patients with severe sepsis are indicated by asterisks.

A metric that we have developed to quantify global transcriptional changes over a pre-determined baseline was used to further investigate the source of heterogeneity in the sepsis patient signature (molecular distance; see Materials and methods for details). Cumulative distances from the uninfected control baseline increased progressively from region R2 to regions R4 and R5 of the training set (Figure 4a), and from region R6 to regions R8, R9 and R10 of the test set 1 (Figure 4b). As indicated on the same graphs we also observed that most fatalities occurred in patients found in regions R5 and R9. Septic patients who died showed multiple organ dysfunction when compared to those who survived (Figure 3a, b). The number of patients with severe sepsis was higher in region R5 compared to regions R2 and R4 (86%, 40%, and 40%, respectively; Figure 4a). Most patients with pneumonia, whether due to melioidosis or other organisms, were also in R5 (Figure 3a). Similarly, the number of patients with severe sepsis increased from region R8 (25%) to R9 (67%) in test set 1 (Figure 4b). Despite all patient samples being obtained within 48 hours of the diagnosis of sepsis, these results suggest that the heterogeneity of the blood transcriptional profiles observed among patients with sepsis may be linked to differences in degrees of disease severity.

### Blood transcriptional profiles of septic patients are heterogeneous

Recently, our group has developed a transcriptional module-based analysis that provides pre-determined annotations through literature profiling of sets of functionally related transcripts [27]. This data dimension reduction approach groups transcripts according to similarities in expression pattern in the blood of patients across a wide range of diseases. Focusing the analysis on sets of coordinately expressed transcripts facilitates functional interpretation of the data, with the activity of annotated modules mapped on a standardized grid format. Furthermore, this approach proved robust in comparisons carried out across different microarray platforms [28].

**Figure 5:**
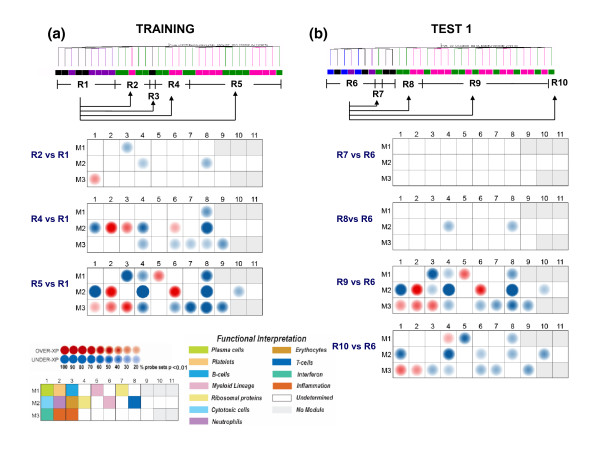
Modular transcriptional fingerprints for regions defined by unsupervised condition clustering. A modular analysis framework was used to generate modular transcriptional fingerprints for the major regions identified in Figure 2. Significant differences in expression levels in comparison to a baseline sample are indicated by a spot, with the intensity of the spot representing the proportion of significantly differentially expressed transcripts for each one of the transcriptional modules. The color of the spot indicates the direction of change of expression: red = overexpressed, blue = underexpressed. For the training set, region R1 was used as the baseline for all comparisons, while for the first test set region R6 was used as the baseline. Functional interpretations are indicated by the color coded grid at the bottom left of the figure.

To facilitate the biological interpretation of the distinct sepsis signatures identified in the present study, we applied this modular analysis strategy. Briefly, differences in expression levels between uninfected controls (region R1) and septic patients (regions R2, R4 or R5) for sets of coordinately expressed transcripts (that is, modules) are displayed on a grid (Figure 5). Each position on the grid is assigned to a given module; a red spot indicates an increase in expression level and a blue spot a decrease. The spot intensity is determined by the proportion of transcripts reaching significance for a given module (≥20% of transcripts in a given module differentially expressed compared to the non-infected group, Mann-Whitney U-test *P *< 0.01). *A posteriori *biological interpretation by unbiased literature profiling has linked several modules to immune cells or pathways as indicated by a color code on the figure legend [27]. The modular map thus constructed for region R2 shows modest over-expression of interferon-inducible transcripts (M3.1: *STAT1*, *IFI35*, *GBP1*) and under-expression of transcripts linked to B-cells (M1.3: *EBF*, *BLNK*, *CD72*), ribosomal proteins (M2.4: *ZNF32*, *PEBP1*, *RPL36*), or T-cells (M2.8: *CD96*, *CD5*, *LY9*) (Figure 5a). An increase in the number of altered modules and spot intensities was observed when comparing region R4 to the uninfected control region (R1), thereby confirming the increased level of perturbation quantified through the earlier computation of cumulative distances (Figure 4). A pronounced over-expression of transcripts associated with neutrophils (M2.2: *BPI*, *DEFA4*, *CEACAM8*), myeloid lineage cells (M2.6: *PA1L2*, *FCER1G*, *SIPA1L2*), and erythrocytes (M2.3: *ERAF*, *EPB49*, *MXI1*) was observed, together with the under-expression of modules associated with ribosomal proteins (M2.4), T-cells (M2.8), and cytotoxic cells (M2.1: *CD8B1*, *CD160*, *GZMK*). This set of modules was similarly affected in septic patients belonging to R5, but this time modules composed of interferon-inducible genes (M3.1: *IFITM1*, *PLAC8*, *IFI35*) and genes related to inflammation (M3.2: *ICAM1*, *STX11*, *BCL3*; M3.3: *ASAH1*, *TDRD9*, *SERPINB1*) were also over-expressed. Modular mapping carried out in turn for our first test set revealed a fingerprint for R9 that was most similar to R5, with both interferon and inflammation-related modules turned on. As described above, we observed that grouping of samples in regions R5 and R9 appeared to correlate with severity of septic illness. Increased abundance of transcripts associated with innate immune responses, including neutrophils, interferon, inflammation, and myeloid lineage, together with under expression of transcripts related to T-cells, B-cells, and cytotoxic cells, indicated substantial dysregulation of the host immune system in response to infection in those patients. This finding is in line with a recent report that found over-expression of transcripts corresponding to inflammation and innate immunity in the blood of patients with sepsis, while the abundance of transcripts related to adaptive immunity was decreased [29]. An interactive version of the module maps shown in Figure 5 is available online [30].

Neutrophils play a pivotal role in the defense against infections. In the present study, over-expression of genes related to this cell type (module M2.2) was observed in septic patients compared to uninfected controls (Figure S4 in Additional data file 2). Increase in transcript abundance for genes included in this module may be an indication of an increase in the abundance of immature neutrophils (for example, *DEFA1*, *DEFA3*, *FALL-39*) as was reported earlier in patients with systemic lupus erythematosus [27,31]. In particular, genes encoding neutrophil cell surface markers, such as *ITGAM *(*CD11b*), *FCGR1 *(*CD64*), *CD62L*, and *CSF3R*, were also over-expressed in septic patients and may be indicative of the activation status of neutrophils.

On the basis of the increased transcriptional perturbation seen in the blood of patients with severe sepsis (regions R4, R5, R9), as shown by both molecular cumulative distance and modular mapping analyses, we interpret the heterogeneity of the sepsis signatures as resulting from differences in levels of disease severity rather than differences in etiology. Longitudinal studies will have to be carried out in order to definitively address this point. We have in addition identified qualitative differences among the transcriptional fingerprints of patients with sepsis corresponding to distinct molecular phenotypes.

### Discovery and validation of a candidate biomarker signature for the diagnosis of septicemic melioidosis

**Figure 6:**
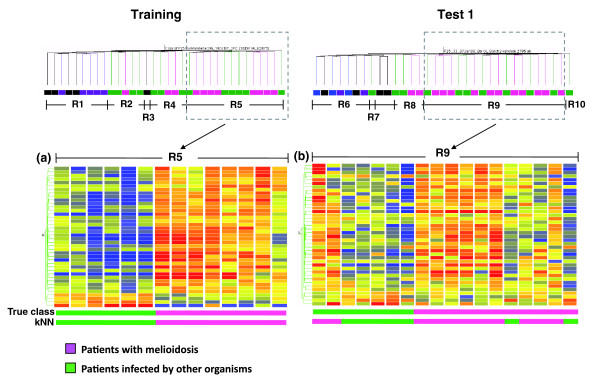
Candidate blood transcriptional markers discriminate sepsis due to *B. pseudomallei *from sepsis due to other organisms. **(a) **Patients with sepsis in R5 of the training set (comprising eight patients with melioidosis (pink rectangles) and six patients with sepsis caused by other organisms (green rectangles)) were subjected to class prediction analysis (K-nearest neighbors (kNN)) using the leave-one-out cross-validation scheme. This algorithm identified 37 classifiers that discriminated samples with 100% accuracy in the training set. **(b) **Independent validation of the 37 predictors was performed with the equivalent region R9 in test set 1, including 11 patients with melioidosis (pink) and 7 patients with sepsis caused by other organisms (green). The predictors correctly classified 14 of the 18 samples (78% accuracy).

**Figure 7:**
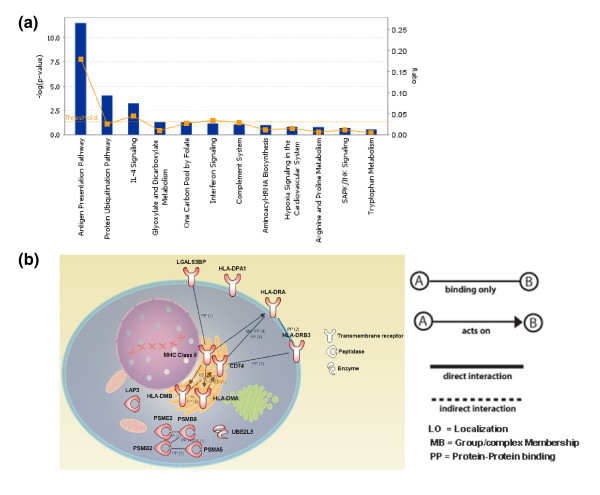
Canonical pathway and gene network analysis of the 37 classifiers. **(a) **The 37 classifiers were analyzed using ingenuity pathway analysis and the classifiers were grouped to 12 canonical biological process pathways. The antigen presentation pathway (7 molecules) and protein ubiquitination pathway (5 molecules) were found to be the dominant canonical pathways represented by these set of classifiers. The orange squares indicate the ratio of the number of genes from the dataset that map to the canonical pathway, whilst the solid blue bars correspond to the *P*-value representing the probability that the association between the genes in the classifier set and the identified pathway occurs by chance alone (calculated by Fischer's exact test, and given as a log *P*-value). A representative gene network of the dominant canonical pathways was then generated **(b)**. Transcripts that are overexpressed in patients with melioidosis are indicated by a red color. The function of the gene product is represented by a symbol. Connections between the gene products and the nature of these interactions are shown.

We focused our biomarker discovery efforts on the prototypical signatures of sepsis established in both training and test sets. Samples clustering in R5 were used for the discovery of a diagnostic signature that distinguishes sepsis caused by *B. pseudomallei *from sepsis caused by other organisms. Class prediction identified a set of 37 classifiers that separated samples from the training set (R5; n = 14) with 100% accuracy in a leave-one-out cross-validation scheme (Figure 6a; K-nearest neighbors at cutoff *P*-value ratio = 0.9 and number of neighbors = 5). Next, the performance of this set of 37 candidate markers was evaluated independently. Samples from region R9 (n = 18) were classified with 78% accuracy (82% sensitivity and 71% specificity; Figure 6b; K-nearest neighbors), with two melioidosis samples and two samples from patients with other infection being incorrectly classified (Table S4 in Additional data file 2). The transcripts forming this candidate biomarker signature are listed in Table 5, with 33 transcripts found to be over-expressed in patients with septicemic melioidosis and 4 underexpressed (*IQWD1*, *OLR1*, *AGPAT9*, and *ZNF281*). Antigen processing and presentation is the strongest functional association identified for this set of 37 classifiers (*P *= 1 × 10^-11^, Fischer's exact test; Figure 7a). Some of the transcripts encode antigen processing and presentation (*PSMB8*, *CD74*) via major histocompatibility complex (MHC) class II molecules (*HLA-DMA*, *HLA-DMB*, *HLA-DRA*, *HLA-DRB2*, and *HLA-DPA1*), and the proteasome complex in the ubiquitin-proteasome system (*UBE2L3*, *PSME2*, *PSMB2*, and *PSMB5*) (Figure 7b). Some of the remaining transcripts are involved in proteolysis (*LAP3*, *CFH*, and *OLR1*), the inflammatory response (*APOL3 *and *AIF1*), apoptosis and programmed cell death (*SEPT4*, *ELMO2*, and *ZAK*), cellular metabolic processes (*ZAK*, *ZNF281*, *SSB*, *WARS*, *MSRB2*, *MTHFD2*, *DUSP3*, and *ASPHD2*), or protein transport (*STX11*). *RARRES3 *is involved in negative regulation of cellular process, *LGALS3BP *is related to the immune response, and *MAPBPIP *is associated with the activation of MAPKK activity. Finally, the list also includes genes that have not previously been associated with the immune response (*IQWD1*, *FAM26F*, *C16orf75*, *AGPAT9*, and *C19orf12*).

**Table 5:** The 37 classifiers discriminated sepsis caused by *B. pseudomallei *from those by other organisms

Rank	Abbreviation	Gene name	Gene accession
1	*FAM26F*	Homo sapiens family with sequence similarity 26, member F	[GenBank:NM_001010919.1]
2	*MYOF*	Myoferlin, transcript variant 2	[GenBank:NM_133337.2]
3	*LAP3*	Leucine aminopeptidase 3	[GenBank:NM_015907.2]
4	*HLA-DMA*	Major histocompatibility complex, class II, DM alpha	[GenBank:NM_006120.3]
5	*WARS*	Tryptophanyl-tRNAsynthetase (WRS)	[GenBank:M61715.1]
6	*RARRES3*	Retinoic acid receptor responder (tazarotene induced) 3	[GenBank:NM_004585.3]
7	*HLA-DMB*	Major histocompatibility complex, class II, DM beta	[GenBank:NM_002118.3]
8	*PSME2*	Proteasome (prosome, macropain) activator subunit 2 (PA28 beta)	[GenBank:NM_002818.2]
9	*C19orf12*	Chromosome 19 open reading frame 12, transcript variant 2	[GenBank:NM_031448.3]
10	*HLA-DRA*	Major histocompatibility complex, class II, DR alpha	[GenBank:NM_019111.3]
11	*CD74*	CD74 molecule, major histocompatibility complex, class II invariant chain transcript variant 2	[GenBank:NM_004355.2]
12	IQWD1*	IQ motif and WD repeats 1	[GenBank:BC025262.1]
13	*APOL3*	Apolipoprotein L3	[GenBank:AF305227.1]
14	*DUSP3*	Dual specificity phosphatase 3	[GenBank:BC035701.1]
15	*SEPT4*	Septin 4, transcript variant 1	[GenBank:NM_004574.2]
16	*CFH*	Complement factor H, transcript variant 1	[GenBank:NM_000186.3]
17	*HLA-DPA1*	Major histocompatibility complex, class II, DP alpha 1	[GenBank:NM_033554.2]
18	*AIF1*	Allograft inflammatory factor 1	[GenBank:U19713.1]
19	OLR1*	Oxidized low density lipoprotein (lectin-like) receptor 1	[GenBank:NM_002543.3]
20	*ASPHD2*	Aspartate beta-hydroxylase domain containing 2	[GenBank:NM_020437.4]
21	*LGALS3BP*	Lectin, galactoside-binding, soluble, 3 binding protein	[GenBank:NM_005567.3]
22	*PSMB2*	Proteasome (prosome, macropain) subunit, beta type, 2	[GenBank:NM_002794.3]
23	*TMSB10*	Thymosin beta 10	[GenBank:NM_021103.3]
24	*STX11*	Syntaxin 11	[GenBank:AF044309.1]
25	*ZAK*	Sterile alpha motif and leucine zipper containing kinase AZK, transcript variant 1	[GenBank:NM_016653.2]
26	*PSMB8*	Proteasome (prosome, macropain) subunit, beta type, 8 (large multifunctional peptidase 7), transcript variant 2	[GenBank:NM_148919.3]
27	*MSRB2*	Methionine sulfoxide reductase B2	[GenBank:NM_012228.3]
28	*HLA-DRB3*	Major histocompatibility complex, class II, DR beta 3	[GenBank:BC008987.1]
29	*ELMO2*	Engulfment and cell motility 2, transcript variant 1	[GenBank:NM_133171.3]
30	*SSB*	Sjogren syndrome antigen B (autoantigen La)	[GenBank:NM_003142.3]
31	*UBE2L3*	Ubiquitin-conjugating enzyme UbcH7	[GenBank:AJ000519.1]
32	*C16orf75 *(*MGC24665*)	Chromosome 16 open reading frame 75	[GenBank:BC022427.1]
33	*AGPAT9 *(*HMFN0839*)*	1-Acylglycerol-3-phosphate O-acyltransferase 9	[GenBank:NM_032717.3]
34	*MTHFD2*	Methylenetetrahydrofolate dehydrogenase (NADP+ dependent) 2, methenyltetrahydrofolate cyclohydrolase	[GenBank:NM_006636.3]
35	*PSMA5*	Proteasome (prosome, macropain) subunit, alpha type, 5	[GenBank:NM_002790.2]
36	ZNF281*	Zinc finger DNA binding protein 99 (281)	[GenBank:AF125158.1]
37	*ROBLD3 *(*MAPBPIP*)	Roadblock domain containing 3	[GenBank:BC024190.2]

*Transcripts underexpressed in patients with septicemic melioidosis when compared to sepsis due to other organisms

The results we have obtained were confirmed by quantitative PCR (qPCR) for the top 11 classifiers chosen after ranking the transcripts based on fold change and difference in intensity (Figure S5 in Additional data file 2). Significant correlation (Pearson correlation test, r = 0.57 or higher, *P *< 0.05) was observed between the expression level determined by microarray and by qPCR in the training (n = 24; Figure S5a in Additional data file 2) and test set 1 (n = 23; Figure S5b in Additional data file 2) for all 11 classifiers.

### Secondary validation of the candidate biomarker signature

**Figure 8:**
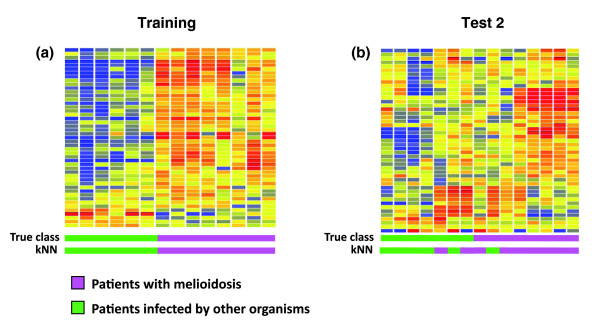
Candidate blood transcriptional markers retain their discriminatory power in an additional secondary validation set. **(a) **Patients with sepsis clustered in region R5 of the training set (comprising eight patients with melioidosis (pink rectangles) and six patients with sepsis caused by other organisms (green rectangles) were hybridized to Illumina Human HT-12 V3 BeadChips and used for microarray analysis as before. The 37 blood transcriptional markers identified from the same samples using Illumina Human V2 BeadChips were used for class prediction analysis of the new dataset in a leave-one-out cross-validation scheme as before. The 37 classifiers discriminated training set samples analyzed using the novel data with 100% accuracy as before, despite the change of microarray platform. **(b) **The performance of the 37 predictor genes was then further evaluated in a secondary independent test set also analyzed using Illumina Human HT-12 V3 BeadChips. This second independent test set (n = 15) comprised eight patients with melioidosis (pink rectangles) and seven patients with sepsis caused by other organisms (green rectangles). The predictors correctly classified 12 of the 15 samples (80% accuracy).

The performance of the candidate biomarkers identified in the training set was further evaluated in a second independent set of samples (n = 15). This secondary validation was performed using the most recent Illumina expression BeadChip (HumanHT-12 V3). The content of this BeadChip was revised to account for updates made to the National Center for Biotechnology Information Reference Sequence database (NCBI RefSeq) since the release of the version 2 BeadChip. We first generated technical replicates by running the cRNA samples of septic patients in region R5 (n = 14) of our training set on the new BeadChip platform. The set of 37 candidate biomarkers identified from analysis using the Hu6 V2 beadchip (40 probes) were mapped to 47 equivalent probes on the HumanHT-12 V3 BeadChip. Class prediction analysis using these 47 probes classified perfectly samples from patients with septicemic melioidosis and patients with sepsis caused by other organisms (region R5 of the training set; 100% accuracy; leave-one-out cross-validation; Figure 8a).

This same set of 47 V3 BeadChip probes was then used to classify the 15 samples of the second test set. Consistent with the results obtained in our first test set, the candidate biomarkers efficiently distinguished patients with septicemic melioidosis (n = 8) from those patients with other pathogens (n = 7) with 80% accuracy (Fisher's exact test, *P*-value = 0.0406) and 3 samples were misclassified (Figure 8b; Table S4 in Additional data file 2). The resulting sensitivity and specificity was 0.71 (exact 95% confidence interval, 0.29 to 0.96) and 0.88 (exact 95% confidence interval, 0.47 to 0.997), respectively.

Thus, class prediction analysis identified and independently validated a candidate blood transcriptional signature for the differential diagnosis of septicemic melioidosis. Furthermore, significant functional convergence was observed among the transcripts forming this signature, which appear to be principally involved in antigen processing and presentation. In the present study, we aimed to compare the signatures of patients with septicemic melioidosis and of patients with sepsis caused by other infections with the goal of identifying candidate biomarkers for the differential diagnosis of melioidosis.

## Discussion

Genome-wide blood transcriptional profiling affords a comprehensive assessment of the immune status of patients. To date, signatures have been reported for a number of systemic diseases, including sepsis [18,22-25,31-35]. A recent report described blood leukocyte mRNA profiles of 35 genes related to inflammation, such as interleukin-1β, interferon-γ, and tumor necrosis factor-α, in patients with melioidosis and healthy control subjects [36]. We have extended the findings of this study with the characterization and independent validation of a robust whole blood signature measured on a genome-wide scale (>48,000 probes) in control subjects and in patients with sepsis caused by a wide range of organisms, including *B. pseudomallei*. Whereas all patients with sepsis clearly demonstrated patterns of expression distinct from that of non-infected controls with over 8,000 transcripts found to be differentially expressed, unsupervised analyses also revealed heterogeneity among the sepsis signature. Applying a modular analysis framework demonstrated differences at the functional level and a molecular distance metric showed marked differences in the levels of transcriptional perturbations between the different patient clusters. We and others have formerly demonstrated pathogen-specific transcriptional signatures in patients with acute infections, but differences in disease etiology could not explain the heterogeneous signatures observed here. These observations support that the first order of variation in this dataset may originate from differences in disease severity. Longitudinal analyses on samples collected serially should be performed to confirm this hypothesis.

A number of studies have employed gene expression microarrays to measure the responses of host cells to pathogenic microorganisms [19-25,37,38]. Specifically, the analysis of patients' blood leukocyte transcriptional profiles has led to a better understanding of host-pathogen interaction and pathogenesis and yielded distinct diagnostic signatures [37-39]. Moreover, others have shown that clinical illness caused by non-infectious causes of systemic inflammatory response syndrome or infection-proven sepsis can be distinguished using the transcriptional signature of peripheral blood mononuclear cells [40]. In addition, illness severity levels and septic shock subclasses of pediatric patients have also been identified through genome-wide expression profiling [41].

Here we report a signature differentiating melioidosis from sepsis caused by other pathogens. Prediction of melioidosis from sepsis caused by other organisms yielded 100%, 78%, and 80% accuracy in the training set and the first and second independent test sets, respectively. The two misclassified patients who were erroneously predicted to belong to the melioidosis group had clinical diagnoses of coagulase-negative staphylococcal (patient I016) and *E. coli *(patient I023) septicemia. Patient I023 had community-acquired septicemia resulting from a leg wound. Patient I016 was hospitalized for 2 weeks prior to the collection of the blood culture from which the coagulase-negative staphylococci were isolated and thus it is plausible that they had true hospital-acquired coagulase-negative staphylococcal septicemia. However, it is equally likely that this isolate was not the true causative agent for the sepsis, in which case it is less surprising that the classification of this sample is incorrect. Coagulase-negative staphylococci were felt to be the organism responsible for sepsis in at least one patient (I018), who was a chronic renal failure patient on dialysis. Coagulase-negative staphylococcal bacteremia is more common in such patients due to the need for frequent connection to plastic lines for dialysis [42]. The organism was isolated in two separate sets of blood cultures from this patient, who was then treated with vancomycin and recovered. For other patients with coagulase-negative staphylococcal bacteremia (I020, I022), the organism was also isolated from two separate sets of blood cultures, suggesting that, in these cases, coagulase-negative staphylococci may be the true causative pathogen. In the remaining cases, it is possible that the coagulase-negative staphylococci were not the true causative pathogen, but the patients meet the criteria for sepsis and thus still form a useful control group against melioidosis, essentially as a group of patients with 'sepsis of uncertain origin'. This reflects a common and important clinical scenario.

Due to concerns over this possible diagnostic misclassification, however, a second independent test set, with no coagulase-negative staphylococcal bacteremia cases, was also used to validate the findings of the training set. Notably, this study adds a second level of validation that goes beyond the training/independent testing scheme that is starting to appear more commonly in microarray publications. The level of classification accuracy of 80% observed in our second independent test set confirmed our earlier results. In this last set two patients with sepsis attributed to *Corynebacterium *spp. (patient I028) and *S. aureus *(patient I029) were misclassified as septicemic melioidosis. These patients stayed in a hospital for more than 10 days before collection of the subsequently positive blood culture. One patient with septicemic melioidosis was erroneously classified as having sepsis caused by another pathogen (patient M033).

We report that the 37 classifiers forming the diagnostic signature were significantly enriched in transcripts whose products are involved in class II antigen processing and presentation, including nonclassical MHC molecules *HLA-DMA *and *HLA-DMB*, which catalyze the removal of invariant chain CD74 from the MHC class II binding groove and facilitate peptide loading to MHC class II molecules within intracellular compartments, as well as classical MHC class II molecules *HLA-DRA*, -*DRB3*, and -*DPA1*, which function by the presentation of loading peptides onto the cell surface. Association between HLA-DRB1*1602 and severe melioidosis in the Thai population has been proposed [43]. Indeed, patients who do not survive sepsis have decreased *HLA-DRA*, *-DMA*, *-DMB*, and *CD74 *mRNA expression in whole blood and reduced *HLA-DR *expression on the cell surface of circulating monocytes [44,45]. The numbers of circulating blood dendritic cells has recently been linked to disease severity in septic patients [46]. This study found significantly lower blood myeloid dendritic cell and plasmacytoid dendritic cell counts in septic patients than in controls. Moreover, decreased numbers of circulating of myeloid and plasmacytoid dendritic cells has also been reported to be associated with mortality in patients with septic shock [46]. Since HLA-DR is a well recognized marker of dendritic cell activation, such findings suggest a possible link between *HLA-DR *expression level, the number of circulating dendritic cells and disease severity. In the present study, decreased mRNA expression of these transcripts was observed in septic patients compared to uninfected controls. Among septic patients, elevated MHC class II mRNA expression discriminated septicemic melioidosis from other sepsis. A recent study has reported decreased expression of these MHC class II molecules in patients with sepsis [29]. Taken together, measuring the expression of these molecules at the transcriptional or protein levels may be useful for the diagnosis of melioidosis. Transcripts encoding the 20S proteasome (*PSMB2*, *PSMB8*, *PSMA5*), 11S activator (*PSME2*) and *UBE2L3 *in the ubiquitin-proteasome pathway, which are responsible for protein degradation and generating pathogen-derived peptides for loading onto MHC class I molecules for presentation to CD8^+ ^T cells, were also listed as classifiers for the differential diagnosis of melioidosis in the present study. The differential expression of transcripts in this pathway has also been reported in patients with dengue hemorrhagic fever [20]. This pathway is believed to be important in host defense against intracellular pathogens and viruses [47]. Given that *B. pseudomallei *is an intracellular pathogen, it is biologically plausible that this pathway would have an important role in the host response to melioidosis. Other classifiers found in our study are also involved in immune responses. Increased abundance of *AIM2 *(interferon-inducible and neutrophil-related gene), *LAP3 *(interferon-inducible gene) and *WARS *(interferon-response gene) found in our study has also been observed to be over-expressed in patients with malaria [19]. These transcripts are induced by interferon-γ, which correlated with our observation of increased abundance of interferon-induced mRNA transcripts (Figure 5, module M3.1) Over-expression of *LGALS3BP*, which is involved in cell-cell and cell-matrix interaction, was also found in our study. Over-expression of this transcript has been reported in the blood of patients with febrile respiratory illnesses and protein levels have been found to be elevated in the serum of patients with human immunodeficiency virus infection [21,48]. The fact that a significant functional convergence exists among the transcripts forming this diagnostic biomarker signature is important as it suggests that it may be stemming from differences rooted in the pathophysiology of *B. pseudomallei*.

In addition to providing valuable diagnostic information, blood transcriptional assays that measure the host response to infection could potentially serve to monitor disease progression and response to treatment. A test combining such characteristics would contribute to improvements in the management of sepsis. In a context where medical care facilities could be quickly overwhelmed, a test measuring the host response to infection would facilitate early diagnosis and an evaluation of disease severity, thus proving to be particularly valuable as a triage tool.

Thus far, several practical considerations have limited the implementation of blood transcriptional testing. Microarray technologies, while constituting an excellent tool in the discovery phase, are currently inadequate for routine testing. Indeed, the data that are generated are not quantitative and are therefore susceptible to batch-to-batch variations. Furthermore, the turnaround time for the processing of samples and generation of data is too long to be of use in a critical care setting. Real-time PCR based assays address such limitations but are only amenable to the quantification of a small number of transcripts. New technologies, however, are becoming available for quantitative 'digital' transcriptional profiling of large sets of genes [49]. An additional advantage of this study is that our findings are based on whole blood transcriptional profiling. This obviates the need for complex additional processing of the blood sample to extract peripheral blood mononuclear cells or other cell fractions or subpopulations, which requires significant laboratory experience and additional equipment. Taken together, the convergence of recent advances made in the collection of blood samples, measurement of transcript abundance and bioinformatics analyses could make clinical translation achievable.

## Conclusions

Microarrays were used to study genome-wide blood transcriptional profiles of patients with sepsis caused by *B. pseudomallei*. We are reporting the identification of a candidate signature for the differential diagnosis of septicemic melioidosis that classified samples with nearly 80% accuracy in a first independent test set and 80% in a second validation set. The molecular distance metric that we describe here for the first time also remains to be evaluated as a potential indicator of disease severity. Finally, the diagnostic signature that we have identified was significantly enriched in genes involved in the MHC class II antigen processing and presentation pathway and the implication of this finding for *B. pseudomallei *pathogenesis will be the subject of further investigations.

## Materials and methods

### Enrolment, sample collection, and informed consent

A total of 598 subjects consisting of 29 uninfected controls and 569 patients suspected of having contracted community-acquired or nosocomial infection were recruited for this study. Of these subjects, those from whom samples were collected in 2006 and met the enrolment criteria were assigned to the training set whereas those from whom samples were collected in 2007 and 2008 were assigned to test set 1 and test set 2, respectively. Clinical specimens (for example, blood, sputum, urine) were collected for bacterial culture within 24 hours following the diagnosis of sepsis. All blood samples were obtained at the Khon Kaen Regional Hospital, Khon Kaen, Thailand. Each patient enrolled in the study had three milliliters of whole blood collected into Tempus vacutainer tubes (Applied Biosystems, Foster City, CA, USA) containing an RNA stabilization solution. The tubes were mixed vigorously for 30 seconds to ensure complete sample homogenization. The whole blood lysate was stored at -80°C prior to extraction. Sixty-three of the enrolled patients had the diagnosis of bacteremic sepsis retrospectively confirmed by the isolation of a causative organism on blood culture. Patients who had negative blood cultures were excluded from further study. Community-acquired septicemia was defined when the first positive blood culture was obtained from samples collected within 48 hours of hospitalization, whereas nosocomial septicemia was defined if the infection developed after 48 hours of hospitalization or within 14 days of a previous admission [50].

The diagnosis of sepsis for this study was taken from accepted international guidelines and defined as presentation with two or more of the following criteria for the systemic inflammatory response syndrome: fever (temperature >38°C or <36°C), tachycardia (heart rate >90 beats/minute), leukocytosis or leukocytopenia (white blood cell count ≥12 × 10^9^/l or ≤4 × 10^9^/l) [51]. Severe infection was defined as the presence of systemic hypoperfusion: shock (systolic blood pressure <90 mmHg or requirement for vasopressors or inotropes for >1 hour in the absence of other causes of hypotension), renal dysfunction (oliguria: urine output <500 ml per 24 hours), liver dysfunction (bilirubin level of >2.0 mg/dl), and thrombocytopenia (platelet count <100,000 cells/ml). A total of 92 blood samples from control subjects and septicemic patients that met the case definitions were analyzed, including 63 patients with sepsis (32 patients with septicemic melioidosis, 31 patients with sepsis due to other organisms) and 29 non-infected controls (9 patients recovered from melioidosis, 12 patients with T2D, and 8 healthy donors) (Figure 1). Among the sepsis group, 3 whole blood samples were collected before antibiotics were given while 60 whole blood samples were drawn after the start of antibiotic therapy. Two samples were collected after anti-fungal drugs were given. Of 32 patients with melioidosis, 20 (63%) had pneumonia, a common clinical presentation of the disease. Twelve patients infected by other organisms also had pneumonia (39%). Clinical information is available in Additional data file 1. The study protocol was approved by the Institutional Review Boards of each participating institution and informed consent was obtained for all subjects.

### Microarray assay

#### RNA preparation and microarray hybridization

Total RNA was isolated from whole blood lysate using the Tempus Spin Isolation kit (Applied Biosystems) according to the manufacturer's instructions. RNA integrity values were assessed on an Agilent 2100 Bioanalyzer (Agilent, Palo Alto, CA, USA). Samples with RNA integrity values >6 were retained for further processing (average RNA integrity values = 7.9, standard deviation = 0.89). Globin mRNA was depleted from a portion of each total RNA sample using the GLOBINclear™-Human kit (Ambion, Austin, TX, USA). Globin-reduced RNA was amplified and labeled using the Illumina TotalPrep RNA Amplification Kit (Ambion). Labeled cRNA was hybridized overnight to Sentrix Human-6 V2 or HumanHT-12 V3 expression BeadChip array (IIlumina, San Diego, CA, USA), washed, blocked, stained and scanned on an Illumina BeadStation 500 following the manufacturer's protocols. The dataset described in this manuscript is deposited in the NCBI Gene Expression Omnibus (GEO) with series accession number [GEO:GSE13015].

#### Microarray data extraction and normalization

##### Microarray data analysis

###### Normalization

Illumina's BeadStudio version 2 software was used to generate signal intensity values from the scans. After background subtraction, the average normalization recommended by the BeadStudio 2.0 software was used to rescale the difference in overall intensity to the median average intensity for all samples across multiple arrays and chips. After that, the standard normalization procedure for one-color array data in GeneSpring GX7.3 software (Agilent Technologies) was used. In brief, data transformation was corrected for low signal, with intensity values < 10 set to 10. In addition, per-gene normalization was applied by dividing each probe intensity by the median intensity value for all samples.

###### Unsupervised analysis

The aim is to group samples on the basis of their molecular profiles without *a priori *knowledge of the phenotypic classification. The first step consists in selecting transcripts that are expressed in the dataset, and present some degree of variability: transcripts must have a detection *P*-value less than the *P*-value cutoff of 0.01 in at least two samples (data file filter in GeneSpring GX 7.3); and they must vary by at least two-fold from the median intensity calculated across all samples with a minimum difference ≥200. The probes passing the filtering criteria were used to group samples in GeneSpring GX 7.3 following two distinct strategies, hierarchical clustering and PCA.

Hierarchical clustering is an iteratively agglomerative clustering method that was performed to find similar transcriptional expression patterns and to produce gene trees or condition trees representing those similarities. The hierarchical clustering performed in our dataset was calculated through the average linkage while the similarity or dissimilarity of gene expression profiles was measured using Pearson correlation, which is the default in the software. By using this algorithm, samples were segregated into distinct groups based on similarity in expression patterns. Gene trees are represented in the horizontal dimension while condition trees are represented in the vertical dimension. The color conventions for all maps are as follows: red indicates over-expressed transcripts, blue underexpressed transcripts, and yellow transcripts that do not deviate from the median.

PCA on conditions was performed to visualize the differences in expression levels of the entire dataset. This approach was performed through JMP genomics software (SAS, Cary, NC, USA) to find and interpret the complex relationships between variables in the dataset from each study group. The first three components, PC1, PC2 and PC3, were plotted against each other. Each colored dot represents an individual sample.

###### Supervised analysis

The aim of the supervised analysis is to identify probes that are differentially expressed between study groups and that might serve as classifiers. We adopted two different strategies for probe selection. First, transcripts that were present in at least two samples in the dataset were selected for statistical group comparison. Second, the parametric Welch *t*-test was used with *P *< 0.01 and three levels of stringency for multiple testing correction - Bonferroni, Benjamini and Hochberg, and no multiple testing correction were set for the statistical group comparison (GeneSpring GX 7.3 software).

###### Class prediction

Class prediction analyses were carried out to determine whether whole blood from patients with sepsis due to *B. pseudomallei *infection carry gene expression signatures that can classify them separately from that of whole blood obtained from septic patients caused by other organisms. Significantly different transcripts (parametric Welch *t*-test, *P *< 0.01) changing by at least 1.5-fold between the study groups were used as a starting point for the identification of classifiers using the K-nearest neighbors algorithm. This set of classifier genes was validated in an independent group of patients (test sets 1 and 2).

###### Molecular distance analysis

This novel approach consists in the computation of a score representing the 'molecular distance' of a given sample relative to a baseline (for example, healthy controls). This approach essentially consists in carrying out outlier analyses on a gene-by-gene basis, where the dispersion of the expression values found in the baseline samples (controls) is used to determine whether the expression value of a single case sample lies inside or outside two standard deviations of the controls' mean. This analysis was performed by merging the transcripts from all modules, which accounted for 2,109 probes. The distance of each sample from the uninfected control baseline was calculated as follows. In step 1 the baseline is established: for each gene the average expression level and standard deviation of the uninfected control group is calculated. In step 2 the 'distance' of an individual gene from the baseline is calculated: the difference in raw expression level from the baseline average of a gene is determined for a given sample, and then the number of standard deviations from baseline levels that the difference in expression represents is calculated. In step 3 filters are applied: qualifying genes must differ from the average baseline expression by at least 200 and 2 standard deviations. In step 4 a global distance from baseline is calculated: the number of standard deviations for all qualifying genes is added to yield a single value, the global distance of the sample from the baseline.

###### Transcriptional module-based analysis

This mining strategy has been described in detail elsewhere [27]. Briefly, a total of 139 blood leukocyte gene expression profiles were generated using Affymetrix U133A&B GeneChips (44,760 probe sets). Transcriptional data were obtained for eight experimental groups, including systemic onset juvenile idiopathic arthritis, systematic lupus erythematosus, liver transplant recipients, melanoma patients, and patients with acute infections (*E. coli*, *S. aureus*, and influenza A). For each group, transcripts with an absent flag call across all conditions were filtered out. The remaining genes were distributed among 30 sets by hierarchical clustering (k-means algorithm; clusters C1 through C30). The cluster assignment for each gene was recorded in a table and distribution patterns across the eight diseases were compared among all the genes. Modules were selected using an iterative process starting with the largest set of genes that belonged to the same cluster in all study groups (that is, genes that were found in the same cluster in eight of the eight groups). The selection was then expanded to include genes with 7 of 8, 6 of 8 and 5 of 8 matches to the core reference pattern. The resulting set of genes from each core reference pattern formed a transcriptional module and was withdrawn from the selection pool. The process was repeated starting with the second largest group of genes, then the third, and so on. This analysis led to the identification of 5,348 transcripts that were distributed among 28 modules. Each module was attributed a unique identifier indicating the round and order of selection (for example, M3.1 was the first module identified in the third round of selection). In the context of the present study, RefSeq IDs were used to match probes between the Affymetrix U133 and Illumina Hu6 platforms. Unambiguous matches were found for 2,109 out of the 5,348 Affymetrix probe sets.

##### RT-PCR and qPCR

RNA expression of a selection of the predictor genes was determined by qPCR. The same source of RNA used for microarray analysis was reverse-transcribed in a 96-well plate using the High Capacity cDNA Archive kit (Applied Biosystems, San Diego, CA, USA). Real-time PCR was set up with Roche Probes Master reagents and Universal Probe Library hydrolysis probes (Roche Applied Science, Indianapolis, IN, USA). PCRs were performed on the LightCycler 480 (Roche Applied Science). Secondary derivative calculation data were collected and cross point values of the selected predictor genes were normalized to two housekeeping genes (*HRPT1 *and *TBP*) [52]. Relative Expression Software Tool (REST^©^) was used in analyzing both group comparison and individual fold changes [53]. Primer sequences were as follows: *ZAK *[GenBank:NM_016653.2] forward primer, 5'-tgacagagcagtccaacacc-3', and reverse primer, 5'-acacatcgtcttccgtccat-3'; *FAM26F *[GenBank:NM_001010919.1] forward primer, 5'-ttctgcagctgaaattctgg-3', and reverse primer, 5'-tgcatgctctgtggctttac-3'; *LAP3 *[GenBank:NM_015907.2] forward primer, 5'-gctggaaagctgagagagactt-3', and reverse primer, 5'-cctgatgcagaccataaaagg-3'; *HLA-DMA *[GenBank:NM_006120.3] forward primer, 5'-agctgcgctgctacagatg-3', and reverse primer, 5'-tggccacattggagtagga-3'; *MYOF *[GenBank:NM_133337.2] forward primer, 5'-agcacgtggaaacaaggact-3', and reverse primer, 5'-ccacccacatctgaagttttc-3'; *WARS *[GenBank:M61715.1] forward primer, 5'-cattttcggcttcactgaca-3', and reverse primer, 5'-gggaatgagttgctgaagga-3'; *RARRES3 *[GenBank:NM_004585.3] forward primer, 5'-tgggccctgtatataggagatg-3', and reverse primer, 5'-ggactgagaagacactggagga-3'; *HLA-DMB *[GenBank:NM_002118.3] forward primer, 5'-gcccttctggggatcact-3', and reverse primer, 5'-tggttttggctacttgcaca-3'; *PSME2 *[GenBank:NM_002818.2] forward primer, 5'-gggaatgagaaagtcctgtcc-3', and reverse primer, 5'-tcaatcttggggatcaggtg-3'; *HLA-DRA *[GenBank:NM_019111.3] forward primer, 5'-caagggattgcgcaaaag-3', and reverse primer, 5'aagcagaagtttcttcagtgatctt-3'; *LGALS3BP *[GenBank:NM_005567.3] forward primer, 5'-tgtggtctgcaccaatgaa-3', and reverse primer, 5'-ccgctggctgtcaaagat-3'.

## Abbreviations

MHC: major histocompatibility complex; NIAID: National Institute of Allergy and Infectious Diseases; PC: principal component; PCA: principal component analysis; qPCR: quantitative PCR; T2D: type 2 diabetes.

## Authors' contributions

RP designed the research, performed the research, analyzed the data, and wrote the paper; SB supported the clinical data; MB wrote the paper; DB performed statistical analysis; GJB designed the research; JB designed the research; GL designed the research; and DC designed the research, analyzed the data, and wrote the paper.

## Additional data files

The following additional data are available with the online version of this paper: a table containing specific information regarding individual patients enrolled in this study (Additional data file 1); a document containing Figures S1 to S5 and Tables S1 to S4 (Additional data file 2).

## Supplementary Material

Additional data file 1 Specific information regarding individual patients enrolled in this study. Click here for file

Additional data file 2 Figure S1 shows the results from a PCA based on 2,785 genes that passed the filtering criteria of 2-fold change and 200 differences from the raw intensity of individual patients when compared to the median intensity across all samples. Figure S2 represents the Gene Ontology term enrichment analysis of 2,785 transcripts forming the unsupervised hierarchical clustering heatmap shown in Figure 2. Figure S3 shows genes that are differentially expressed between septic patients and uninfected controls. Figure S4 shows blood transcriptional expression profiles of neutrophil-related genes in patients with sepsis when compared to uninfected controls. Figure S5 shows linear regression and correlation coefficients of the expression signals obtained from qPCR and microarray analyses. Table S1 lists the hematological data from all patients. Table S2 lists the genes with significant differences in expression between patients with sepsis and uninfected controls. Table S3 lists predictor genes that differentiate septic patients from non-infected controls. Table S4 shows the summary of class prediction analysis. Click here for file
