# Supplementary material for: Genomic transcriptional profiling identifies a candidate blood biomarker signature for the diagnosis of septicemic melioidosis
Source: Genome Biol. 2009 Nov 10;10(11):R127. doi: 10.1186/gb-2009-10-11-r127 (PMC3091321; doi:10.1186/gb-2009-10-11-r127)
Supplement: Additional data file 2 — Figure S1 shows the results from a PCA based on 2,785 genes that passed the filtering criteria of 2-fold change and 200 differences from the raw intensity of individual patients when compared to the median intensity across all samples. Figure S2 represents the Gene Ontology term enrichment analysis of 2,785 transcripts forming the unsupervised hierarchical clustering heatmap shown in Figure 2. Figure S3 shows genes that are differentially expressed between septic patients and uninfected controls. Figure S4 shows blood transcriptional expression profiles of neutrophil-related genes in patients with sepsis when compared to uninfected controls. Figure S5 shows linear regression and correlation coefficients of the expression signals obtained from qPCR and microarray analyses. Table S1 lists the hematological data from all patients. Table S2 lists the genes with significant differences in expression between patients with sepsis and uninfected controls. Table S3 lists predictor genes that differentiate septic patients from non-infected controls. Table S4 shows the summary of class prediction analysis. [file gb-2009-10-11-r127-S2.DOCX]

**Supporting Information**

**Genomic Transcriptional Profiling Identifies a Candidate Blood Biomarker Signature for the Diagnosis of Septicemic Melioidosis**

Rungnapa Pankla, Surachat Buddhisa, Matthew Berry, Derek M Blankenship, Gregory J Bancroft, Jacques Banchereau, Ganjana Lertmemongkolchai, Damien Chaussabel.

**I. Supplementary Figures**

**
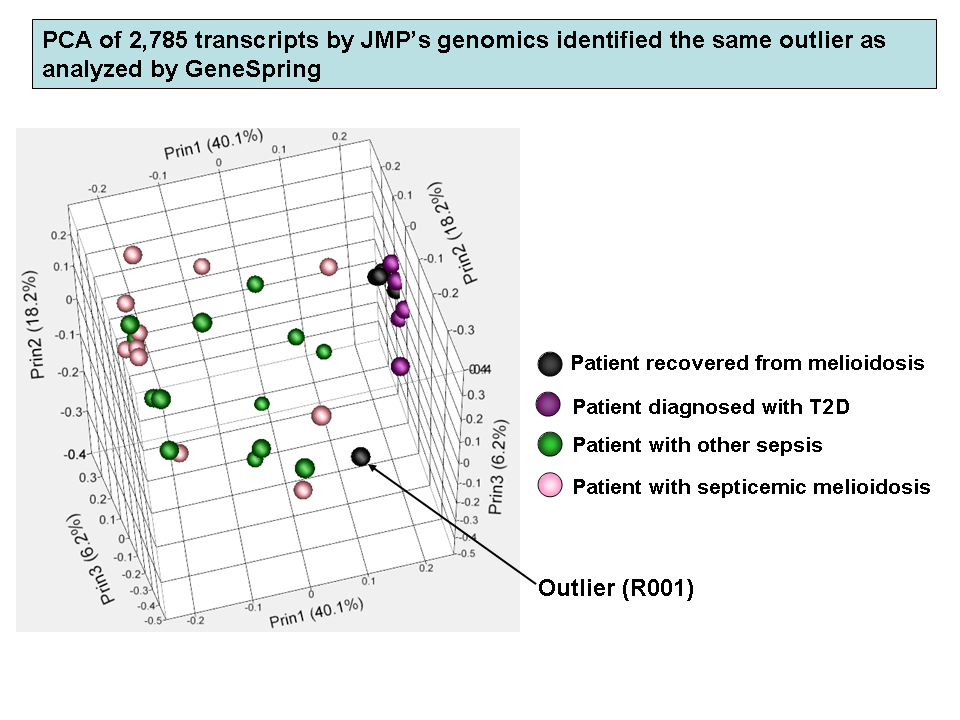
**

**Figure S1. Principal component analysis.**

The 2,785 genes passing the filtering criteria as described in figure 2 were used to generate a PCA condition plot. Three-dimensional projections of PC1 (40.1%), PC2 (18.2%) and PC3 (6.2%) accounting for 64.5% of the total variation is rendered. Patients with septicemic melioidosis are indicated in pink (n=11), patients with sepsis caused by other pathogens in green (n=13), patients recovered from melioidosis and patients with T2D (controls, 5 subjects each group) are in black and purple, respectively. One outlier (R001) was excluded from subsequent analysis on the basis of this PCA.

.


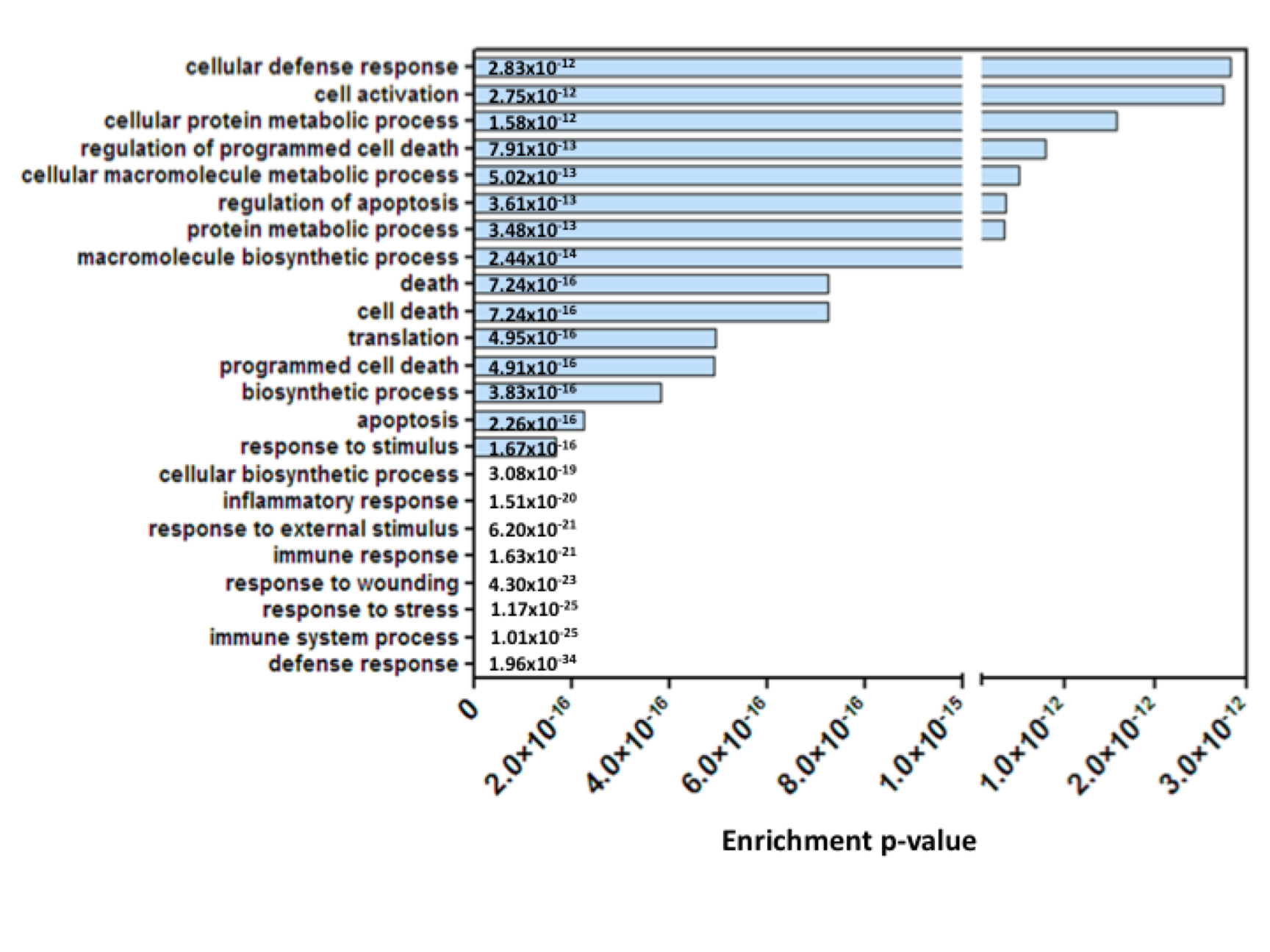


**Figure S2. Gene Ontology Term enrichment analysis.**

Functional enrichment of 2,785 transcripts forming the unsupervised hierarchical clustering heatmap figure 2 were analyzed by the **D**atabase for **A**nnotation, **V**isualization and **I**ntegrated **D**iscovery (DAVID)/**E**xpression **A**nalysis **S**ystematic **E**xplorer (EASE). The top enrichment score of GO terms were at p-value <0.05 with Benjamini-Hochberg false discovery rate of 5% were indicated.


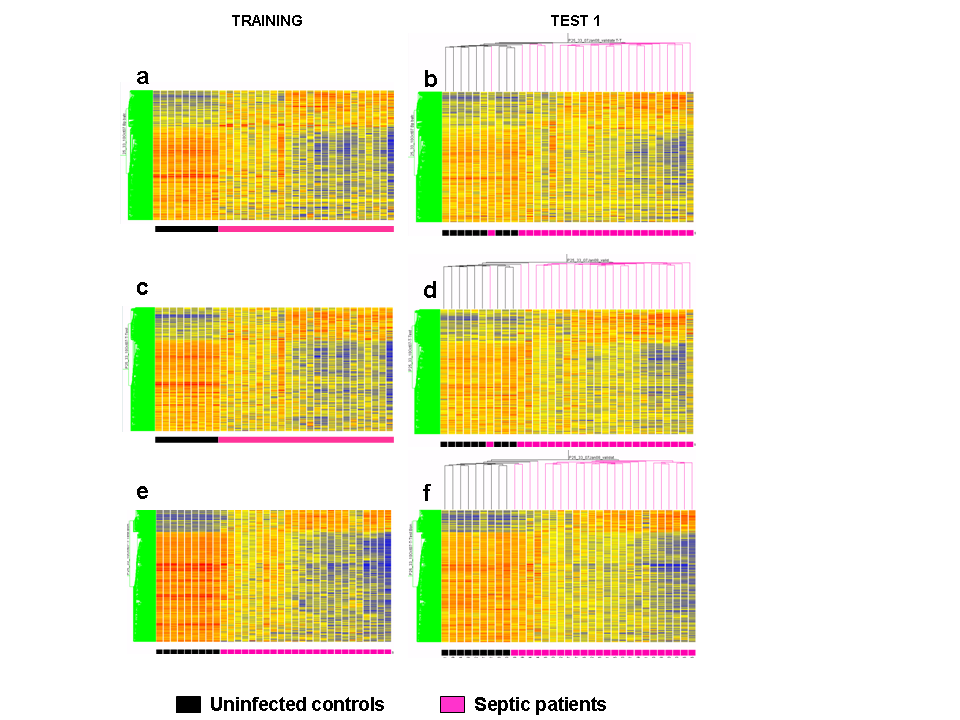


**Figure S3. Class comparison analysis.**

Statistical testing identified transcripts differentially expressed between septic patients (pink, n=24) and controls (black, n=9) groups in the training set (Welch t-test, p<0.01). Comparisons were carried out in the absence of multiple testing correction (8,096 transcripts), and when using Benjamini and Hochberg (7,377 transcripts), and the Bonferroni correction (2,733 transcripts), with resulting heatmaps shown in **a**, **c**, and **e**, respectively. Hierarchical condition clustering was used to order samples in the independent test set 1 (24 septic patients and 9 uninfected controls) using the list of transcripts and gene trees obtained in the training set: without correction **(b)**, as well as with Benjamini and Hochberg **(d)** or Bonferroni correction **(f)**.


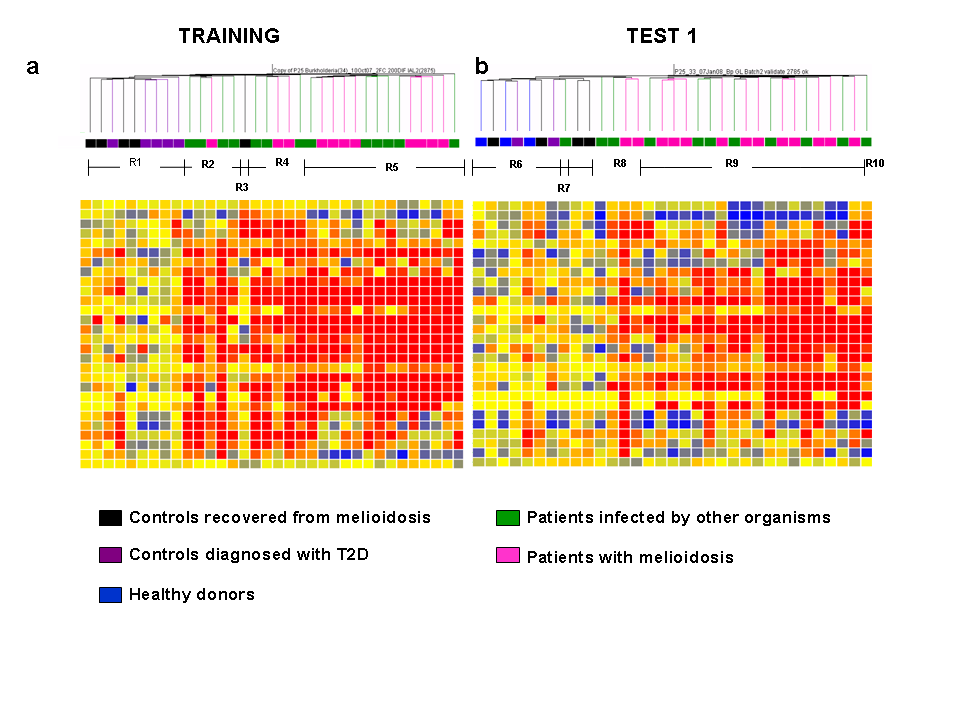


**Figure S4. Expression profiles of neutrophil-related genes module M2.2.**

The 28 transcripts forming module M2.2 were clustered after normalization to region R1 of the training set **(a)** or R6 for the test set 1 **(b)** (Uninfected controls). Samples were ordered according to regions defined in Figure 2.

**a**


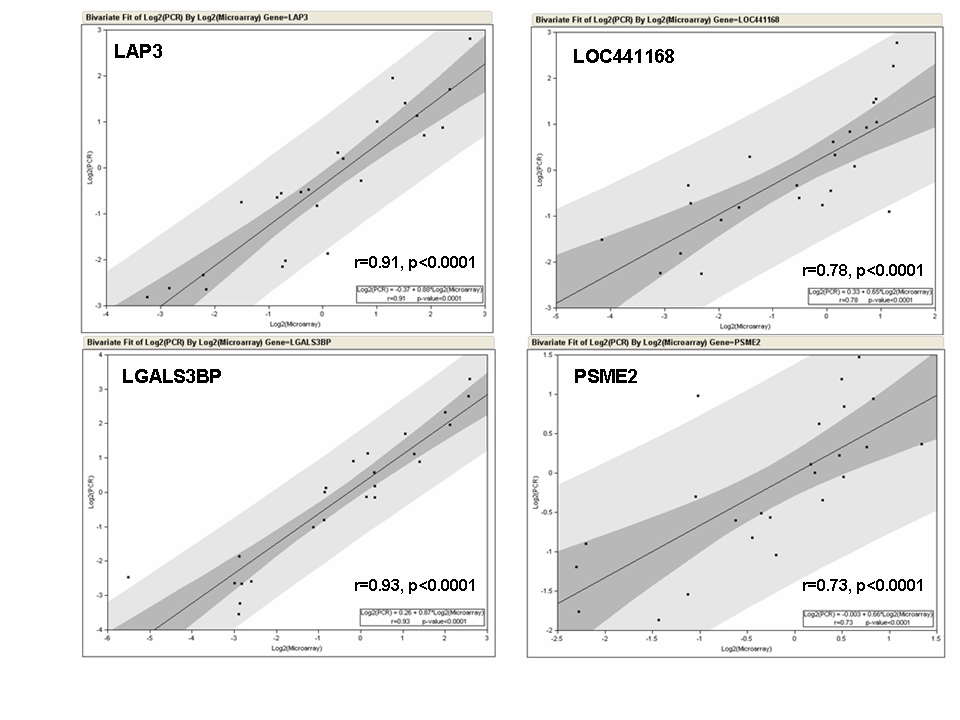

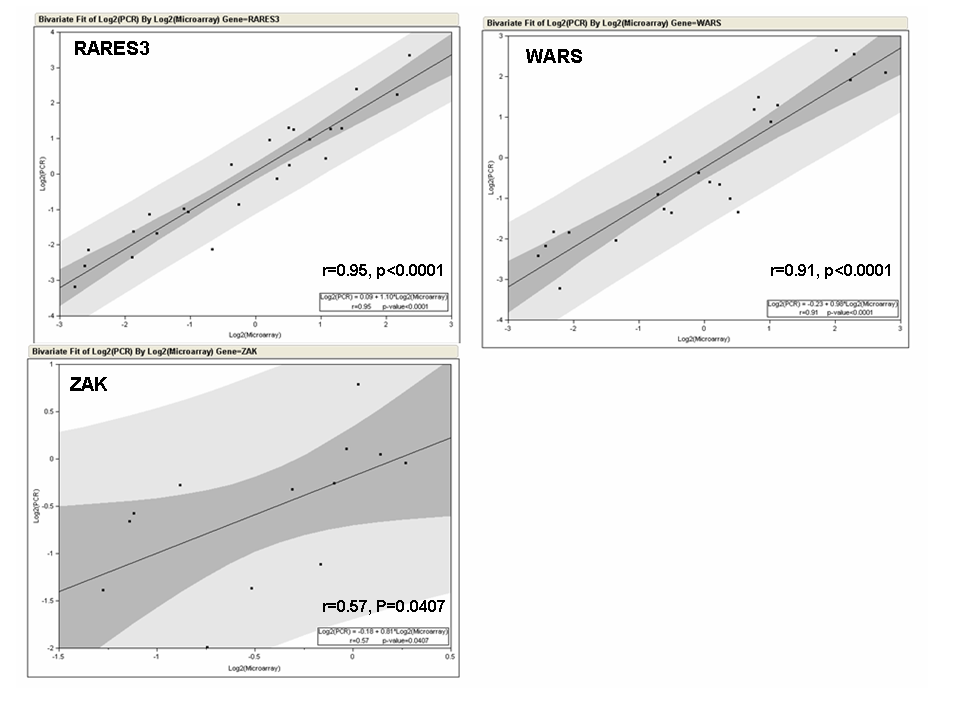

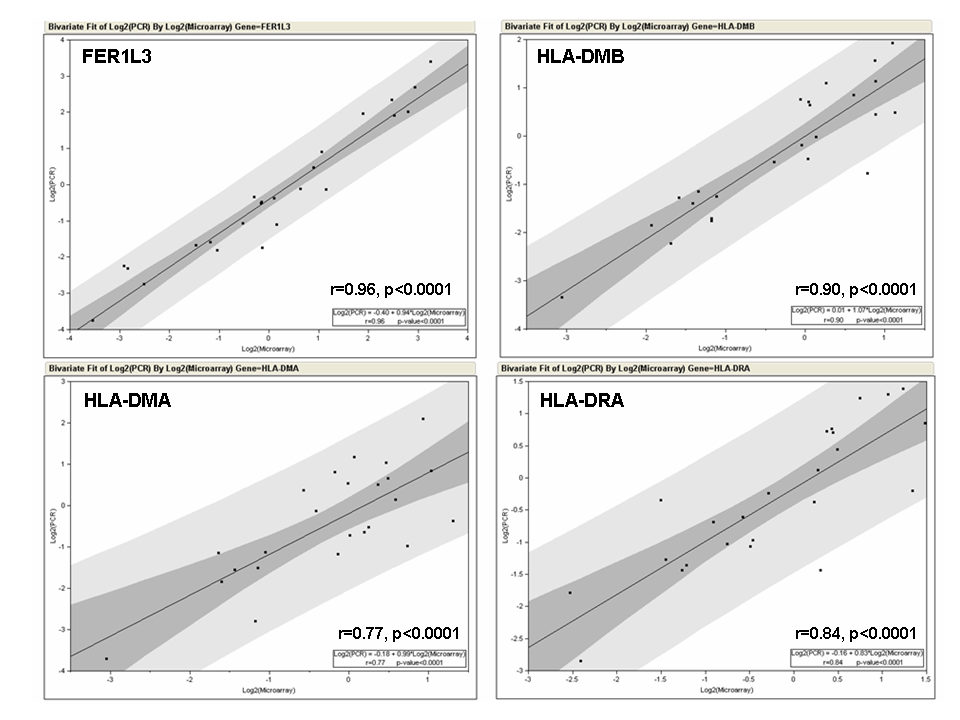


**b**


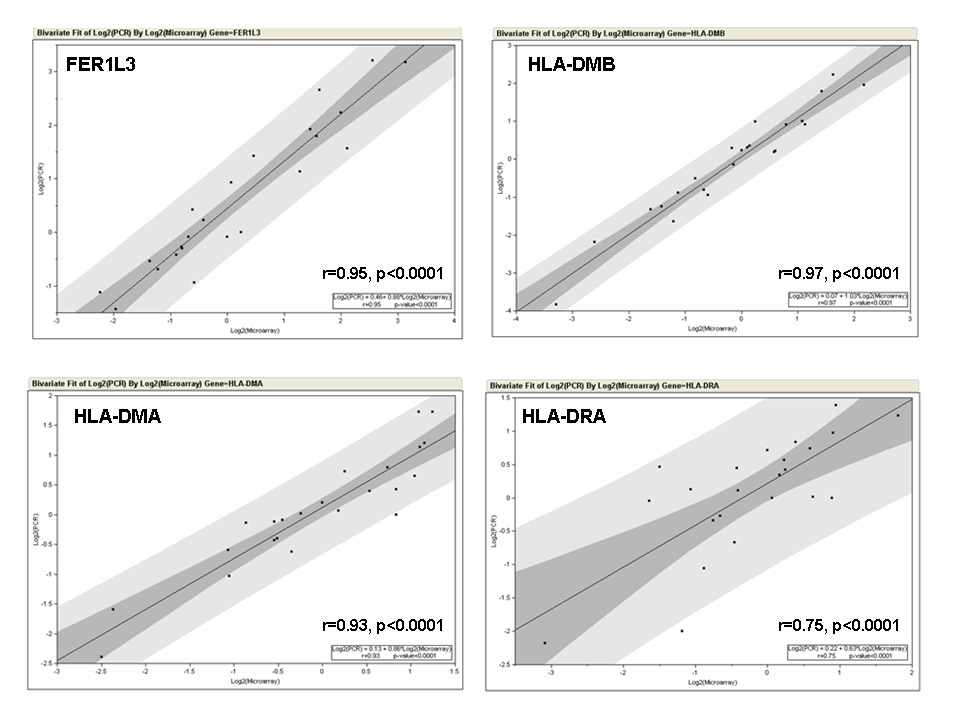

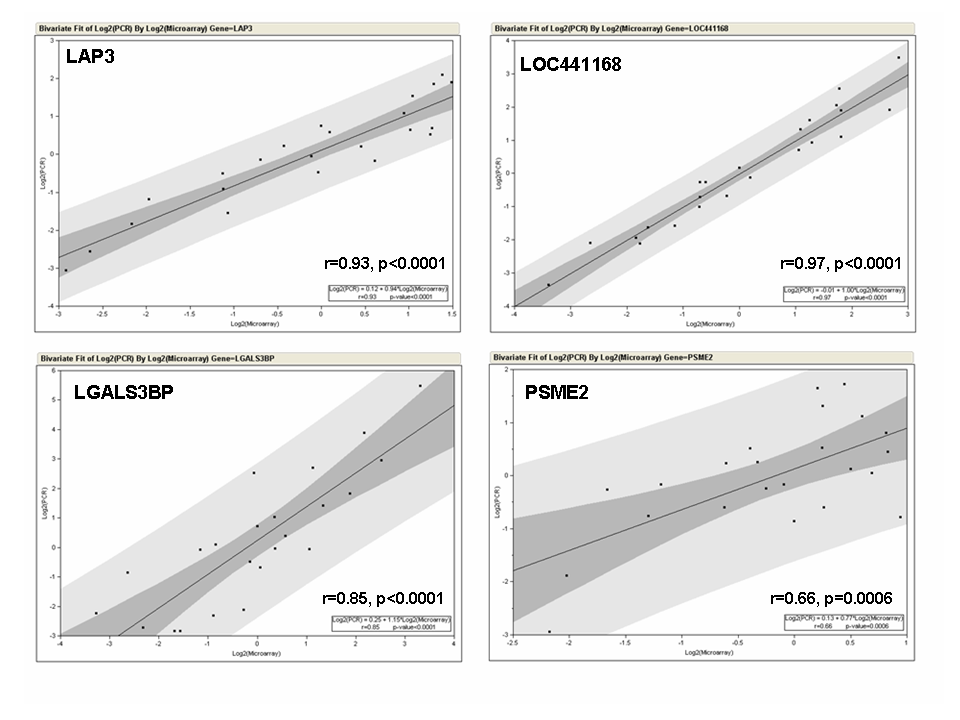

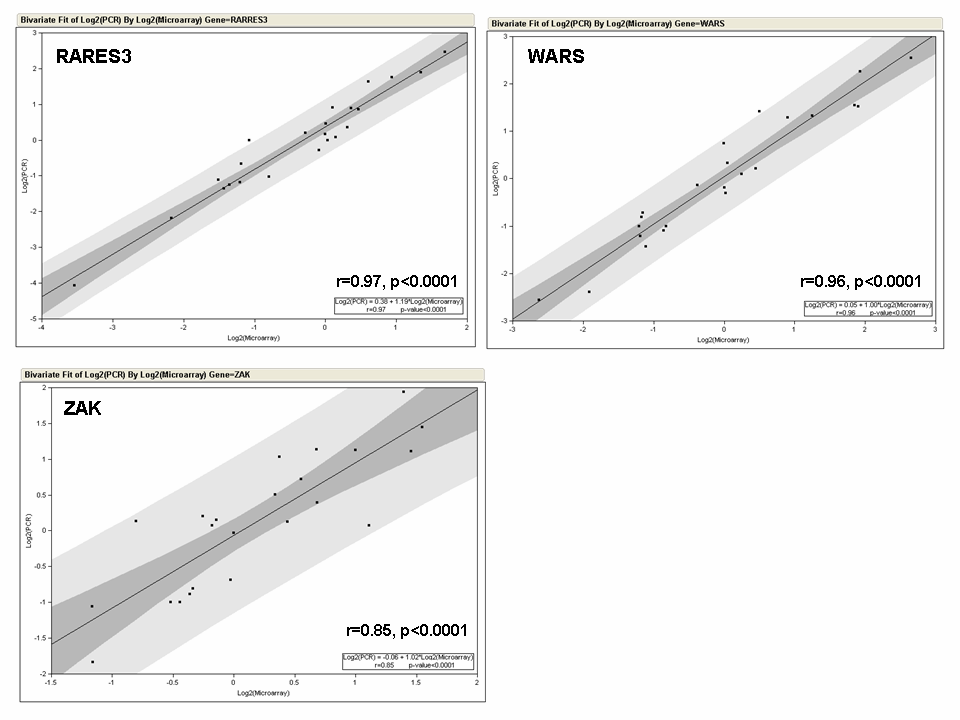


**Figure S5. Correlation of qPCR and microarray gene expression data**.

Eleven classifiers were selected for a confirmatory qPCR assay. Transcript abundance was measured for the training set including 13 patients with sepsis due to other pathogens and 11 patients with septicemic melioidosis **(a)** and the test set 1 including 10 patients with sepsis caused by other pathogens and 13 patients with melioidosis **(b)**. qPCR and microarray expression values were normalized to the median across all samples in each training (n=24) or test set 1 (n=23). qPCR and microarray expression values for each individual gene were correlated. Linear regression and Pearson correlation coefficients are given for each transcript. The light shaded area indicates the 95% confidence limits for individual predicted values. The dark shaded area indicates the 95% confidence limits for the slope and intercept.

.

**II. Supplementary tables**

**Table S1. Differential white blood cells and platelet counts**

| **Cell types (Normal range 10^9^/l)** | **Groups** | **Datasets** | **Mean (StDev)** | ***F* value** | **p-value^a^** |
| --- | --- | --- | --- | --- | --- |
| Leukocytes (4.5-10.0) | Other sepsis | Training (n=13) | 11.7 (7.5) | 0.64 | 0.6685 |
|  |  | Test set 1 (n=11) | 14.5 (9.2) |  |  |
|  |  | Test set 2 (n=7) | 12.8 (5.1) |  |  |
|  | Septicemic Melioidosis | Training (n=11) | 13.3 (6.6) |  |  |
|  |  | Test set 1 (n=13) | 11.2 (6.6) |  |  |
|  |  | Test set 2 (n=8) | 17.1 (13.9) |  |  |
| Lymphocytes (1.5-3.0) | Other sepsis | Training (n=13) | 1.1 (0.7) | 0.33 | 0.8959 |
|  |  | Test set 1 (n=11) | 1.5 (1.1) |  |  |
|  |  | Test set 2 (n=7) | 1.5 (0.7) |  |  |
|  | Septicemic Melioidosis | Training (n=11) | 1.8 (3.2) |  |  |
|  |  | Test set 1 (n=13) | 1.1 (0.7) |  |  |
|  |  | Test set 2 (n=8) | 1.5 (1.1) |  |  |
| Monocytes (0.3-0.5) | Other sepsis | Training (n=13) | 0.5 (0.7) | 0.75 | 0.5897 |
|  |  | Test set 1 (n=11) | 0.4 (0.5) |  |  |
|  |  | Test set 2 (n=7) | 0.7 (0.6) |  |  |
|  | Septicemic Melioidosis | Training (n=11) | 0.5 (0.4) |  |  |
|  |  | Test set 1 (n=13) | 0.5 (0.4) |  |  |
|  |  | Test set 2 (n=8) | 0.2 (0.2) |  |  |
| Neutrophils (3.15-6.2) | Other sepsis | Training (n=13) | 9.9 (6.6) | 0.63 | 0.6784 |
|  |  | Test set 1 (n=11) | 12.2 (8.0) |  |  |
|  |  | Test set 2 (n=7) | 10.5 (4.6) |  |  |
|  | Septicemic Melioidosis | Training (n=11) | 11.1 (6.6) |  |  |
|  |  | Test set 1 (n=13) | 9.4 (6.1) |  |  |
|  |  | Test set 2 (n=8) | 14.6 (11.7) |  |  |
| Platelets (140-440 ) | Other sepsis | Training (n=13) | 161.7 (135.3) | 0.64 | 0.6666 |
|  |  | Test set 1 (n=11) | 226.6 (121.3) |  |  |
|  |  | Test set 2 (n=7) | 276.1 (218.5) |  |  |
|  | Septicemic Melioidosis | Training (n=11) | 208.4 (140.8) |  |  |
|  |  | Test set 1 (n=13) | 218.1 (137.2) |  |  |
|  |  | Test set 2 (n=8) | 222.9 (107.8) |  |  |

**^a^** ANOVA Overall F test

StDev = Standard deviation

**Table S2. Transcripts significant differences in expression between patients with sepsis when compared to non-infected controls.**

| **Groups** | **Dataset** | **FDR** | **MTC** | **Differential expressed transcripts** |
| --- | --- | --- | --- | --- |
| 24 sepsis  vs  9 controls | 16,400 Transcripts | 1% | No correction | 8,096 Transcripts |
|  |  |  | Benjamini and Hochberg | 7,377 Transcripts |
|  |  |  | Bonferroni | 2,733 Transcripts |

FDR = False Discovery Rate

MTC = Multiple Testing Correction

**Table S3.** **K-nearest neighbors analysis to identify best predictor genes classified septic patients from controls.**

Leave-one-out cross-validation was performed in training set then predictors were validated on the test set 1.

| Number of neighbors | Predictor genes | Training set | | | Test set 1 | | |
| --- | --- | --- | --- | --- | --- | --- | --- |
|  |  | Correct predictions | Incorrect predictions | Not predicted | Correct predictions | Incorrect predictions | Not predicted |
| 6 | 50 | 31 | 2 | 0 | 32 | 1 | 0 |
|  | 40 | 31 | 2 | 0 | 32 | 1 | 0 |
|  | 30 | 31 | 2 | 0 | 32 | 1 | 0 |
|  | 20 | 31 | 2 | 0 | 32 | 1 | 0 |
|  | 10 | 30 | 3 | 0 | 31 | 2 | 0 |
| 5 | 50 | 31 | 2 | 0 | 32 | 1 | 0 |
|  | 40 | 31 | 2 | 0 | 32 | 1 | 0 |
|  | 30 | 31 | 2 | 0 | 32 | 1 | 0 |
|  | 20 | 31 | 2 | 0 | 32 | 1 | 0 |
|  | ***10*** | ***32*** | ***1*** | ***0*** | ***31*** | ***2*** | ***0*** |
| 4 | 50 | 31 | 2 | 0 | 32 | 1 | 0 |
|  | 40 | 31 | 2 | 0 | 31 | 1 | 1 |
|  | 30 | 31 | 2 | 0 | 30 | 1 | 2 |
|  | 20 | 31 | 1 | 1 | 30 | 1 | 2 |
|  | 10 | 30 | 1 | 2 | 30 | 2 | 1 |

**Table S4. Performance of the 37 classifiers on prediction of septicemic melioidosis from other sepsis**

Training set (n=14)

|  | Disease Status | |
| --- | --- | --- |
| Test | Melioidosis | Other sepsis |
| + Melioidosis | 8 | 0 |
| - Melioidosis | 0 | 6 |
| Total | 8 | 6 |

Sensitivity=8/8 (100%) Specificity=6/6 (100%)

Test set 1 (n=18)

|  | Disease Status | |
| --- | --- | --- |
| Test | Melioidosis | Other sepsis |
| + Melioidosis | 9 | 2 |
| - Melioidosis | 2 | 5 |
| Total | 11 | 7 |

Sensitivity=9/11 (82%) Specificity=5/7 (71%)

Fisher’s exact p-value = 0.0491

Power = 0.86

Test set 2 (n=15)

|  | Disease Status | |
| --- | --- | --- |
| Test | Melioidosis | Other sepsis |
| + Melioidosis | 7 | 2 |
| - Melioidosis | 1 | 5 |
| Total | 8 | 7 |

Sensitivity=7/8 (88%) Specificity=5/7 (71%)

Fisher’s exact p-value = 0.0406

Power = 0.80
